# Spindle Oscillations in Sleep Disorders: A Systematic Review

**DOI:** 10.1155/2016/7328725

**Published:** 2016-03-10

**Authors:** Oren M. Weiner, Thien Thanh Dang-Vu

**Affiliations:** ^1^PERFORM Center and Center for Studies in Behavioural Neurobiology, Department of Exercise Science and Department of Psychology, Concordia University, Montréal, QC, Canada H4B 1R6; ^2^Centre de Recherche de l'Institut Universitaire de Gériatrie de Montréal and Department of Neurosciences, Université de Montréal, Montreal, QC, Canada H3W 1W5

## Abstract

Measurement of sleep microarchitecture and neural oscillations is an increasingly popular technique for quantifying EEG sleep activity. Many studies have examined sleep spindle oscillations in sleep-disordered adults; however reviews of this literature are scarce. As such, our overarching aim was to critically review experimental studies examining sleep spindle activity between adults with and without different sleep disorders. Articles were obtained using a systematic methodology with* a priori* criteria. Thirty-seven studies meeting final inclusion criteria were reviewed, with studies grouped across three categories: insomnia, hypersomnias, and sleep-related movement disorders (including parasomnias). Studies of patients with insomnia and sleep-disordered breathing were more abundant relative to other diagnoses. All studies were cross-sectional. Studies were largely inconsistent regarding spindle activity differences between clinical and nonclinical groups, with some reporting greater or less activity, while many others reported no group differences. Stark inconsistencies in sample characteristics (e.g., age range and diagnostic criteria) and methods of analysis (e.g., spindle bandwidth selection, visual detection versus digital filtering, absolute versus relative spectral power, and NREM2 versus NREM3) suggest a need for greater use of event-based detection methods and increased research standardization. Hypotheses regarding the clinical and empirical implications of these findings, and suggestions for potential future studies, are also discussed.

## 1. Introduction

Adult sleep is comprised of non-rapid-eye-movement (NREM) and rapid-eye-movement (REM) states, which alternate approximately every 90 minutes of sleep. NREM sleep is divided into three stages, NREM1, NREM2, and NREM3, which now includes what was earlier separated as NREM stage 4. The proportions of these sleep stages change across the lifespan, and this is partially attributed to developmental changes in multiple neurochemical and hormonal sleep-regulation processes [1]. Boselli and colleagues [2] demonstrated that, compared to teenage or middle-aged participants, elderly subjects spent more time in NREM1 sleep and less time in NREM3 and REM sleep, and had more arousals across sleep stages. These results were corroborated by findings from a meta-analysis of 65 sleep studies from childhood to old age [3].

These different stages of sleep are based on the measurement of rhythmic and/or repetitive neural oscillations during sleep (cf., [4]), which provides a means to examine how distinct brain rhythms relate to patterns of cortical activity across varying states of arousal [5]. Technical and statistical developments have allowed for more detailed examinations of neural oscillations during sleep and advanced the field of sleep research beyond the traditional quantification of time- (e.g., minutes spent in each sleep stage), proportion- (e.g., percentage of REM sleep), or ratio-based sleep variables (e.g., arousal index and rapid eye movement density). Measurement of sleep oscillations can be performed using visual inspection of oscillation events, automatic event detector algorithms, and spectral analysis techniques (each described further below).

During relaxed wakefulness and the transition to sleep, a sinusoidal alpha rhythm is present (8–13 Hz; 20–40 *μ*V) [6]. NREM1, or light sleep, is characterized by slow eye movements, vertex sharp waves (50–200 ms duration, up to 250 *μ*V; [7]), and low amplitude, mixed frequency activity, predominantly in the theta range (4–7 Hz; 50–100 *μ*V; [8]). NREM2 sleep is characterized by the presence of K-complexes and sleep spindles. K-complexes, which can be spontaneous or evoked, are slow, high-amplitude delta waves (0.5–3 Hz, typically 100–400 *μ*V over frontal derivations) lasting at least 0.5 seconds [8, 9]. Sleep spindles (11–15 or 16 Hz; described below) are trains of oscillations which wax and wane in amplitude and last 0.5 to 3 seconds [8]. NREM3, or slow-wave sleep (SWS), is characterized by the presence of slow (0.5–4 Hz), high-amplitude (≥75 *μ*V) delta waves (also called slow wave activity, SWA) in 20% of a given epoch, which reflect cortical synchronization [6, 8], and is associated with sleep homeostasis [10]. REM sleep is characterized by the presence of rapid eye movements (REMs), muscle atonia with occasional transient muscle activity, saw tooth waves (triangular, often serrated, centrally-maximal 2–6 Hz waves), and low amplitude, mixed frequency activity that resembles NREM1 or relaxed wakefulness [8]. REM sleep can be further divided into tonic (no REMs) and phasic (REMs) periods.

Sleep spindles are a characteristic sign of sleep. Spindle activity is attributed to interactions between thalamic reticular, thalamocortical, and cortical pyramidal networks [11, 12]. Spindle sequences occur when rhythmic bursts of inhibitory postsynaptic potentials (IPSP) are transmitted from GABAergic thalamic reticular to glutamatergic thalamocortical neurons. When large and long enough, these IPSPs result in the deinactivation of a low-threshold Ca^2+^ current. This in turn produces spike bursts consisting of fast Na^+^-mediated action potentials, which are transferred to cortical neurons where excitatory postsynaptic potentials (EPSP) lead to action potentials and therefore spindle sequences visible on the electroencephalography (EEG). Cortical neurons then provide excitatory feedback to GABAergic thalamic reticular neurons, which restarts the spindle sequence [13–15].

Spindles can occur during NREM2 and NREM3 sleep [8] and have been observed as time-locked to the depolarization of slow oscillations ([16, 17], see also [18, 19]). Spindle frequencies can be slow (~12–14 Hz, anterior distribution) or fast (~14–16 Hz, posterior distribution), and spindle density fluctuates between high and low within each sleep cycle, typically following a U-shaped curve; spindles also show an initial decrease and subsequent linear increase across successive sleep cycles [14, 18, 20, 21]. The presence of spindles changes dramatically across the lifespan (e.g., infancy, puberty, and old age; see [18, 19] for reviews). The development of spindle oscillations across infancy, childhood, and adolescence warrants special attention given the potential influence of early brain development on neural oscillation activity in adulthood; however, a discussion of pediatric spindles is beyond the scope of this paper. Among adults, Peters et al. [22] demonstrated that spindle density was significantly higher in young (*M*_age_ = 20.75 yrs) compared to older participants (*M*_age_ = 71.17 yrs; *M* [SD] spindle density 3.84 [1.43] versus 1.14 [0.83]), with differences between younger and older adults noted across successive sleep stages and age deciles.

Astori et al. [23] argued that spindle activity contributes to neuronal development (e.g., associations between increased spindle activity and neurodevelopmental milestones), memory consolidation, and maintenance of sleep continuity. Increased spindle activity/density following exposure to a presleep learning task, and positive correlations between spindle parameters and postsleep test performance, have been observed in studies of declarative memory using all-night sleep data (left frontocentral spindles, [24]; bilateral parietal spindles, [25]) and during NREM2 specifically [26, 27]. Greater spindle density and frequency have also been positively associated with performance on motor sequence learning tasks [28, 29] and on verbal and visual attention tasks [30]. The role of spindles in memory consolidation during SWS has also been examined. Mölle and colleagues [17] demonstrated that not only do fast parietal spindles (12–15 Hz) and slow frontal spindles (9–12 Hz) occur at different phases of slow delta oscillations (~0.75 Hz; earlier versus later, resp.), but also prior learning augmented the frequency of these event sequences (fast spindle-slow oscillation-slow spindle), and this increased frequency was positively associated with test performance. Similarly, Cox and colleagues [31] demonstrated that greater spindle density specifically during slow-wave sleep was positively correlated with declarative memory performance. A separate line of research has examined spindles in relation to sleep maintenance and continuity (see [13, 23]). In a study by Dang-Vu and colleagues [32], healthy participants with greater spindle rates on a quiet baseline night required more intense sounds presented on subsequent nights to cause a cortical arousal during NREM2 and during a combined NREM2 + NREM3 sleep state. Besset et al. [33] provided additional support for the sleep continuity hypothesis by demonstrating that, compared to good-sleeper controls, sleep-maintenance insomniacs display attenuated spindle activity overall and a weaker association between the time-course of spindle activity (increasing) and SWA (decreasing) across sleep time. However, this study was limited by a small sample size (*n* = 7 in each group) and is thus not considered further in the present review (see inclusion criteria below). Taken together, studies suggest important dynamics between spindle activity and NREM sleep in promoting cognition and helping to maintain sleep continuity in tandem with declining SWA across sleep time [20, 34]. Considering these spindle functions, the measurement of spindle activity in different populations can offer important insights into brain dysfunction and the pathophysiology of disease. However, accurate spindle measurement requires careful consideration of multiple methodological factors.

The AASM manual indicates that optimal EEG analysis includes data sampled at 500 Hz, with electrode impedances minimized (e.g., 5 KΩ; [8]), and caution is warranted about accurate detection of EEG amplitude vis-à-vis chosen filters and derivations [9]. Silber and colleagues [9] noted that optimal recording of K-complexes and delta activity occurs over frontal derivations, of the alpha rhythm over posterior derivations, and of sleep spindles over central derivations.

Measurement of sleep oscillations is typically performed by visual inspection of EEG sleep events, by automated analysis using event detector algorithms, or by examining spectral power within prespecified frequencies. While visual inspection of sleep oscillation events is generally considered a gold-standard method, this notion has been challenged (e.g., low scoring agreement between sleep-stage transition epochs; [35, 36]). Visual scoring is also time-consuming and can introduce inter- and intrascorer biases (e.g., subjective judgments and visual acuity differences [9]). Automatic detection algorithms reduce subjective biases by increasing reliability and objectivity but are known for occasional problems with differentiating ambiguous oscillation signals (e.g., true signal versus artefact; alpha versus spindles), and are also highly influenced by the algorithm and detector settings chosen by researchers (e.g., signal duration, frequency, and amplitude characteristics) [37, 38].

Automatic spindle detectors come in several forms, many of which have been tested empirically, but none of which are considered “optimal” or “better.” Further, no standard guidelines exist for spindle detector properties, settings, and output, although some have been recently proposed [39]. Huupponen and colleagues [37] compared several spindle detection algorithms in replicating manually scored spindles recorded from 12 healthy adults. The study demonstrated that an automatic detector that considered both the sigma index (a measure indicating the probability of a spindle event based on an amplitude function within a pre-defined bandwidth) and a time-domain measure of spindle amplitude as defining factors replicated visually scored NREM2 spindles (70% true positive, 32% false positive) better than a detector that relied solely on spindle amplitude (70% true positive, 46% false positive). These authors also concluded on the importance of detector flexibility due to intersubject variability in spindle activity. In a subsequent study, Huupponen et al. [40] tested a modified spindle detector that used a fuzzy amplitude threshold estimation method instead of prespecified amplitudes. Performance of the fuzzy detector, which uses Bayesian estimation techniques to produce an optimal threshold for spindle detection, was largely equivalent to that of visual scoring (range of true positive rate/false positive rate: detector [73.4–84.9/2.3–5.4], visual [71–83.6/1.9–5.5]). The authors discussed and concluded on the benefits of detecting individualized amplitude thresholds opposed to using a fixed amplitude value with automatic spindle detectors.

Besides the identification of sleep stages and microstructural oscillation events using visual or automated event detection, spectral analysis techniques (e.g., fast Fourier transform, FFT) provide a continuous measure of the variance (or power) in brain activity during sleep in specific bandwidths [41]. Standard spectral bands in the sleep EEG have been identified (delta [0.25–4 Hz], theta [4–8 Hz], alpha [8–12 Hz], sigma [11–16 Hz, mainly reflecting spindle activity], and beta [>16 Hz]), with power in each band distributed both across and within NREM and REM periods [42, 43]. The degree of power density in each frequency band, commonly expressed in absolute or relative/normalized units, is thought to reflect the activity of various interconnected neural networks that mediate different brain functions [42, 44]. For example, age-related changes in sleep spectral power have been observed [45], with declines in slower frequencies often attributed to reduced sleep need, increased sleep fragmentation, and/or reduced sleep efficiency (e.g., [46]).

Spectral analysis of EEG characterizes rhythmic neural activity during sleep and can also help examine how brain activity during sleep contributes to/reflects the pathophysiology of disease and/or daytime functioning. This technique is advantageous in examining sleep oscillations that are not visually discernable, but results are dependent on several technical specifications (e.g., sampling rate) and researcher-defined variables (e.g., analysis window) and are also sensitive to contamination of EEG signals by even small intrusions of artifact (e.g., EMG; [47]). Moreover, there is wide variability in reported EEG frequency bands across the literature, as well as the reporting of absolute (actual power) and relative spectral measures (proportion of power in one spectral band vis-à-vis all other bands; [42, 48]). These inconsistencies can make it challenging to meaningfully compare results across different studies.

Examining spindle activity using spectral analysis is performed by measuring EEG sigma density, typically quantified as spindle frequency activity (SFA, 12–15 or 16 Hz). One advantage of measuring SFA is that it elucidates the continuous progression of EEG sigma power (i.e., a proxy for spindle activity) across sleep time [46, 49, 50]. SFA has a varying (reciprocal) relationship with SWA across sleep cycles and is also related to sleep homeostasis. Studies show a positive relationship between SFA and SWA at the beginning and end of a NREM cycle, but a negative relationship in the middle (decreased SFA, increased SWA), as well as a linear increase across successive sleep cycles that corresponds with decreases in SWA [46, 50]. The increase in SFA may also be modulated by circadian-based factors influencing SWA power timing [49]. Similar to visually scored spindles, SFA has a frequency-specific topography [51] that may also reflect a stable individual difference [52], and lifespan changes in SFA are evidenced in how older (versus younger) adults have attenuated SFA power overall [45, 46, 53] and a blunted time-course of SFA power across successive sleep cycles [45]. Together, measurement of SFA is useful in characterizing sleep-related pathology (e.g., SFA-SWA dynamics/interactions) and can help corroborate objective and subjective indications of poor sleep.

An increasing number of studies have examined neural oscillations during sleep to better characterize sleep pathology. Aberrant neuronal oscillations during sleep offer insights regarding neurophysiological functioning and network connectivity and allow for hypotheses about the pathophysiology of various illnesses and diseases, as well as possible intervention targets. In particular, examination of sleep spindles has a number of theoretical and clinical implications regarding our understanding of how brain activity during sleep is affected by, and potentially contributes to, the development of sleep disorders.

There is an abundance of studies, mostly with adults, using EEG and polysomnographic (PSG) methods to examine macro- (e.g., sleep architecture) and microlevel (e.g., REM density) sleep variables in clinical participants with various sleep disorders (e.g., see [54] for a meta-analysis of PSG-measured sleep differences in studies comparing insomnia and good-sleeper participants). An increasing number of studies have examined more fine-tuned measures of neural oscillations and sleep microstructure, such as spindle activity, but there appears to be more data available from nonclinical, relative to clinical, populations. Given the popularity of more advanced EEG techniques (e.g., spectral analysis) in studying brain activity during sleep, for example, to help elucidate functional brain differences to aid diagnosis and treatment, a growing body of literature has become available on sleep spindle activity in people with sleep disorders. However, while broad reviews of sleep oscillations in general exist among various sleep-disordered samples (e.g., [42, 55–57]), there is a paucity of literature summarizing research specifically on spindle/sigma activity between nonclinical and clinical groups with various sleep disorders. As such, the primary purpose and overarching aim of this paper is to provide an updated review of studies examining spindles and spindle activity during sleep between healthy and sleep-disordered adults. Beyond reviewing and contrasting findings between various spindle measures and different sleep disorder populations, this review will (a) consider pertinent empirical and clinical implications for distinct activity of spindle oscillations between healthy and clinical groups and (b) critically evaluate research findings and conclusions in relation to study methodology.

## 2. Literature Search Strategy

**Figure 1:**
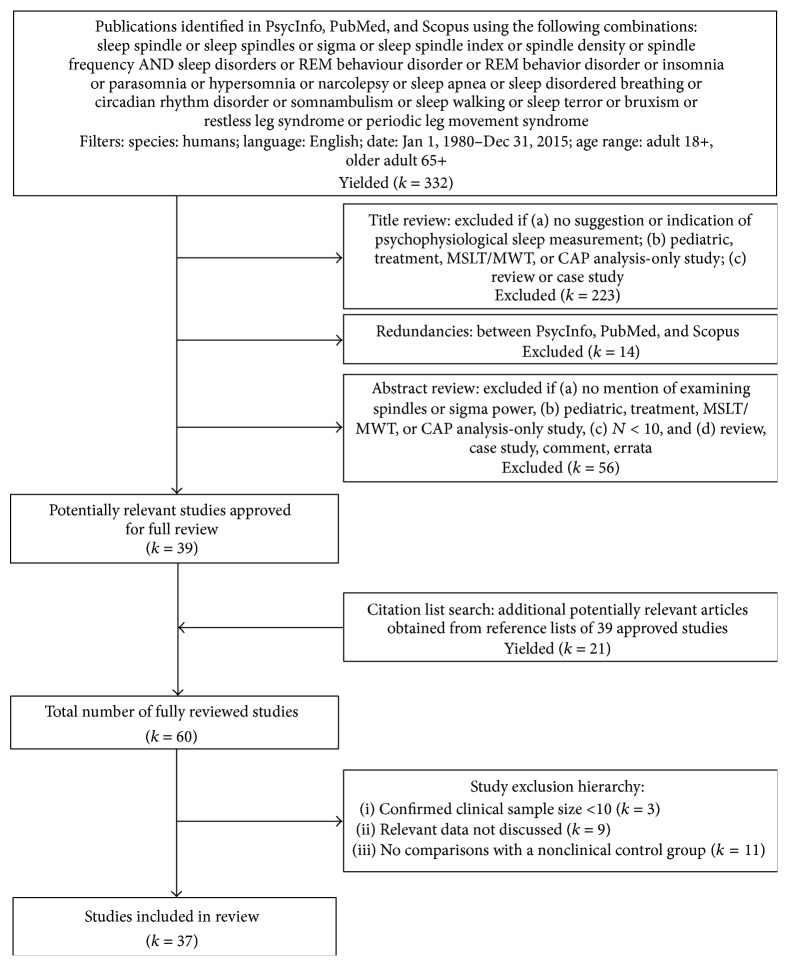
Flow chart for article identification and inclusion.

Articles were obtained using a systematic methodology. Using PsycInfo, PubMed, and Scopus databases, a search was conducted in June 2015 using keywords associated with sleep spindle measures (e.g., spindles, sigma power, and spindle frequency activity) and with different sleep disorder populations (e.g., insomnia, sleepwalking, and narcolepsy; Figure 1). This search yielded 332 hits. Titles were accepted if they indicated or suggested that a psychophysiological sleep study was conducted in participants with a sleep disorder. Titles were rejected if they explicitly indicated that (a) participants were healthy/nonclinical, (b) participants were children or adolescents, (c) the study focused only on MSLT/MWT data or a specific intervention, or (d) the article was a review, case study, chapter, comment, or response. The abstracts of 95 nonredundant titles that were accepted were then reviewed using stringent criteria. Abstracts were selected for further follow-up if they reported obtaining EEG/PSG sleep data from adult participants with a clinical sleep disorder and explicitly identified examining spindles or a spindle-related variable, or examining a broad EEG spectrum. Abstracts were excluded if they reported (a) sample size less than 10, (b) pediatric sample only, (c) healthy/nonclinical group only or did not identify group status (clinical, nonclinical); (d) a pharmacological/psychological intervention study, (e) examining only basic PSG variables (e.g., total sleep time, % in REM sleep, and duration in sleep stages), only activity within a nontarget EEG frequency band (e.g., alpha and beta), only cyclic alternating pattern (CAP), or only other measures (e.g., heart rate and respiration) between sleep stages, (f) measuring and analyzing only MSLT/MWT or sleep actigraphy data, (g) EEG/PSG data used purely for screening or diagnostic confirmation purposes, or (h) a case study, review article, dissertation, or book chapter. Thirty-nine abstracts were accepted and their associated articles were obtained for full review. Additionally, citation lists of the 39 studies accepted for review were scanned for potentially relevant articles not identified in the initial search. Using the same inclusion/exclusion criteria for titles and abstracts described above, the citation search yielded 21 additional articles for evaluation. In total, 60 articles were fully reviewed. Reviewed articles were subsequently excluded if they (a) confirmed a clinical sample size < 10 (*k* = 3), (b) did not report spindle/sigma mean-level or frequency parameters (e.g., studies examining only spindle coherence) or did not report target variables obtained from overnight PSG studies (e.g., MSLT study; *k* = 9), or (c) did not statistically compare target data with a nonclinical control group (*k* = 11). Following these exclusions, 37 studies, organized across several diagnostic categories, were included in this review.

Reviewed studies were organized below by diagnostic category of the study's clinical group (see Tables 1–3). Studies that compared samples with more than one clinical group (e.g., insomnia versus narcolepsy versus controls) were arbitrarily categorised based on the group of primary focus or of first mention in the article/title. Data reported in Tables 1 to 3 reflect summaries of methods and results reported in each article; unless otherwise specified, presented results are study findings that showed statistically significant between-group effects. Articles are organized in three* a priori* categories: insomnia, hypersomnias/disorders of excessive daytime sleepiness, and sleep-related movement disorders/parasomnias. Only one article examined sigma power in patients with bruxism [58]; as there was no other study available to draw comparisons, this study will be briefly considered at the end and discussed only in-text. Following a brief introduction to each sleep disorder category, the review will first broadly characterize the obtained sample of articles before summarizing and examining the research. A primary distinction was made between studies that used visual/automatic spindle detection analyses and studies that used spectral analyses. A brief summary and discussion of the reviewed studies is provided at the end of each section.

Across the following review, four overarching observations about the available literature on spindle oscillations in sleep disorders will become apparent. First, there exist many more studies among patients with insomnia and sleep-disordered breathing than among patients with narcolepsy/cataplexy, idiopathic hypersomnia, restless legs syndrome, parasomnias, and bruxism. Second, each of the reviewed studies was cross-sectional, which precludes any sound inferences about temporal relations between disturbed sleep and altered spindle activity. Third, reviewed studies largely utilized spectral analysis techniques to characterize spindle oscillations, suggesting a need for more studies using visual- and/or event-based detection methods; further, most spectral analysis studies were not examining spindle activity, specifically. Finally, there are noted between-study discrepancies in sample characteristics and in spindle recording and analysis procedures (e.g., EEG recording sites and spectral bandwidths), which highlight the need for greater methodological standardization in the spindle research field.

## 3. Insomnia

*Insomnia* is a common sleep disorder characterized by difficulties initiating or maintaining sleep despite adequate opportunities for sleep, or with waking up too early, along with daytime impairment in cognitive, emotional, social, and/or vocational functioning [59]. Insomnia has been regarded as a 24-hour disorder of hyperarousal [60] and is associated with multiple pathologies and broader risk factors, including sleep-related breathing and movement disorders, medical or neurological conditions, mental illness, psychosocial stress, and environmental factors [61–63]. Insomnia often coincides with discrepancies between objective (e.g., PSG-derived) and subjective sleep data (e.g., self-reported sleep quality or quantity) [64]. Insomnia can be a primary (i.e., idiopathic, no identifiable factors linked to the disorder's onset and maintenance) or a secondary disorder (i.e., comorbid, occurring alongside psychosocial stress, substance/alcohol use, or medical conditions) and can be further subtyped in psychophysiological (i.e., conditioned sleep difficulties and/or cognitive/physiological hyperarousal in bed) and paradoxical categories (i.e., sleep-state misperception/subjective complaints of poor sleep contrasting with an absence of objective PSG abnormalities [59, 61, 65, 66]). While research diagnostic criteria for insomnia subtypes have been defined [65], variability in definitions across the literature remains. This wide variability in definitions of insomnia subtypes reflects the heterogeneity of the disorder but has also led to sample-based discrepancies in epidemiological research [62].

Fourteen studies that examined spindle activity between participants with insomnia and controls were identified (Table 1). Six of these studies recruited a clinical sample of less than 20 participants, while the remainder recruited 20 or more participants. Sex ratios across the studies varied from 0% to 70% male participants. Importantly, clinical samples represented either a combined or subtyped (e.g., primary or psychophysiological, sleep-maintenance, subjective, or paradoxical) insomnia group, which suggests low homogeneity of clinical samples across the studies. Methodologically, 3 studies examined only one night of EEG data, whereas the rest examined at least one recording obtained after a preliminary adaptation night. Seven studies recorded EEG data using a sampling rate of 256 Hz or greater and 7 studies used a sampling rate of 200 Hz or less. Small but noticeable differences in spindle/sigma frequency bands across studies are also evident. Finally, only one study included a visual analysis of sleep spindles, while the remainder employed spectral analysis. Broadly, observations from this review suggest that spindle oscillations between insomniacs and controls become more evident when subtypes of insomnia (i.e., objective versus subjective) are better defined.

Bastien and colleagues [67] provided the only retrieved study to examine spindles in insomnia using a visual event-detection analysis. The authors initially recruited 16 individuals with chronic primary psychophysiological insomnia and 14 self-defined good sleepers (5 males). Sex proportions in the clinical group are unknown, as two insomniacs were excluded prior to analysis and the authors did not present updated demographic statistics. Using data from 14 participants in each group, the number and density of spindles (12–14 Hz, >0.5 sec duration, 20–40 *μ*V) were analyzed from C3 during NREM2 overall and between early and later NREM2 periods. Insomniacs showed a trend for higher spindle number and density, but no significant group differences were observed. The authors concluded that similar spindle characteristics between the two study groups suggest insomnia sufferers do not evidence greater indications of arousability than controls. Bastien et al. acknowledged the limitations of only examining one spindle bandwidth only during NREM2 and from C3 (versus Cz).

Greater sigma-related activity in insomniacs has been reported among other studies using spectral analysis techniques, albeit somewhat inconsistently. An early study by Freedman [68] recruited a sample of 12 idiopathic sleep-onset insomniacs and 12 controls and examined EEG activity on C3 and O2 from 1 to 30 Hz, measured in 1 Hz bins. Only data from the first artefact-free minute of each sleep stage obtained on a third recording night were analyzed. Freedman reported significantly greater 16 Hz power among insomniacs during the onset of the first REM period on O2. Further, although not statistically significant, insomniacs also had greater power across 1 Hz bins in the sigma band (11–16 Hz) during NREM1 and REM on C3 and O1 and during REM on C3 (14–16 Hz). Freedman's [68] study is limited by only analyzing the first minute of sleep data across stages, not correcting for multiple comparisons, and potential spectral leakage of alpha activity (greater during sleep onset) over occipital derivations, which could explain the lack of significant findings. Finally, given the sex differences found in EEG spectral power (e.g., [79]), it is worth noting that the proportion of males in Freedman's control group was 4 times that of the full insomnia group.

Overall, the comprehensiveness of spectral EEG research in insomnia has developed considerably since Freedman's [68] study, with more recent studies examining all-night sigma activity, and often further comparing data between NREM1–3 and REM and between individual NREM-REM sleep cycles across the night. Merica and colleagues [66] examined sigma (12.5–14.75 Hz) dynamics on F4-Cz across four NREM and four REM sleep cycles. Power in the delta, theta, alpha, and beta bands was similarly examined, although corrections for multiple comparisons were not described. Participants were 20 adults with sleep maintenance insomnia, 17 of whom met criteria for psychophysiological and the remaining 3 for idiopathic insomnia. Both clinical groups were categorized based on International Classification of Sleep Disorders (ICSD) criteria (decreased total sleep time (TST), increased sleep onset latency (SOL), increased periods of waking and NREM1 sleep across sleep time, and low sleep efficiency); however, idiopathic insomnia was considered when sleep problems began in childhood. Importantly, all insomniac patients were still analyzed together. The time-course of absolute sigma power was similar between insomniacs and controls, but the rate of increase and subsequent decrease in sigma across NREM cycles was slower in insomniacs, who also maintained a lower sigma peak during NREM. However, significant differences in sigma power between insomniacs and controls were only observed in the last two NREM cycles. A sustained attenuation of sigma power in insomniacs during the latter half of sleep (i.e., when spindle activity is typically highest and delta power/SWA is lowest) would account for a lower threshold for cortical arousal towards the end of sleep time that can maintain poor/fragmented sleep and morning exhaustion. Conversely, insomniacs displayed significantly higher sigma power across all four REM cycles versus controls, which the authors tentatively interpreted as reflecting NREM and wake intrusions into REM sleep.

Bastien and colleagues [70], Buysse and colleagues [72], and Wu and colleagues [76] each examined absolute sigma power during portions of NREM sleep between primary insomniacs and controls, and all three studies reported no group differences despite noted discrepancies in study methodology (see Table 1). Buysse and colleagues [72] failed to demonstrate overall group differences in spindle power using a conservative (*p* < 0.01) alpha value but reported a significant (*p* < 0.05) group-by-sex interaction during total NREM sleep, wherein greater absolute sigma (12–16 Hz) was evident specifically among female insomniacs. The authors interpreted this finding along with higher spectral power across several bands among female insomniacs as reflecting just one aspect of a generalized increase in EEG power among this group.

Among studies that examined relative sigma power, null findings between insomniacs and controls during NREM sleep periods were a typical result. For instance, five studies (one with an all-female group; [78]) all reported no group differences in relative sigma power (12–14 or 12–16 Hz) across different NREM sleep periods [72, 73, 75, 76, 78]. As well, Staner et al. [71] examined spindle frequency activity (SFA; 11.5–15 Hz) during the first NREM cycle (from sleep onset to REM onset) divided into 10 equal intervals, expressed as a percentage of the median SFA across the entire (undivided) NREM cycle, and reported no differences between age- and sex-matched groups of primary insomniacs, insomniacs with comorbid depression, and controls. Isreal and colleagues [75] recruited 54 primary sleep maintenance insomniacs and reported no differences in relative sigma across three recording nights versus controls. Individual means for each of the three recording nights were not presented, leaving it unclear whether relative sigma activity gradually increased or decreased across successive recordings within each group. However, using intraclass correlations (ICCs), the authors examined the short-term stability of EEG power between groups and observed greater stability of relative sigma across three nights in insomniacs versus controls (ICC = 0.83 versus 0.69). The authors also reported significantly higher alpha and beta activity among insomniacs, and both measures were more stable versus controls. Taken together, these findings are compatible with the authors' argument that heightened cortical arousal may persist in insomnia regardless of extraneous factors, whereas in good sleepers the potential for cortical arousal during sleep may be more a function of circumstance [75]. However, this remains speculative given the lack of group differences in sigma power and also requires further examination using a longer study follow-up duration (e.g., weeks or months).

Among the above-reviewed studies that largely show nonsignificant group differences in sigma power, one common thread between them is that insomnia participants were, in general, not as carefully subdivided into distinct diagnostic categories (i.e., psychophysiological versus paradoxical; objective versus subjective) as they were in studies that showed more group differences. However, variability in results across these studies remains evident. Krystal and colleagues [69] examined absolute and relative sigma power in 30 older patients with persistent primary sleep-maintenance insomnia during a combined NREM2/NREM3 state and during REM sleep. While no group differences were found during REM sleep, relative sigma was greater during NREM2/NREM3 sleep in insomniacs overall. The authors then divided insomniacs into “subjective” and “objective” subtypes based on whether or not a “normal” single-night PSG recording was obtained (normal PSG = minimum 6 hours of total sleep time (TST), >80% sleep efficiency). Results showed that subjective insomniacs (with chronic sleep complaints despite a normal PSG) had significantly higher absolute and relative sigma power during NREM2/NREM3 overall versus objective insomniacs, who in turn had significantly higher sigma power than controls. Next, the authors examined if their results were robust to different criteria used for insomnia subtypes by conducting an iterative reanalysis of NREM EEG power using cut-off scores of “total sleep time” (TST) in 10-minute segments (280–410 min), and percent of sleep time underestimated in 5% segments (−25–25%). Here, “objective” insomniacs were those with TST and percentage of sleep underestimation below each cut-off, and “subjective” insomniacs were those with TST or percentage of sleep underestimation greater than or equal to each cut-off. Subjective insomniacs had significantly higher relative sigma power versus controls in each case at *p* < 0.05. Subjective insomniacs had higher sigma power versus objective insomniacs across several, but not all, sleep time cut-offs at *p* < 0.05. Subjective insomniacs had higher sigma power versus objective insomniacs among sleep time underestimation cut-offs within −10–5%, but only at *p* < 0.10. Objective insomniacs had significantly (*p* < 0.05) higher relative sigma versus controls only with NREM sleep time ≥ 330 minutes, but higher sigma power across all sleep time underestimation cut-offs. The authors noted that analyses were controlled for age and sex and that initial multivariate omnibus tests were used to help minimize Type I error with subsequent follow-up analysis. No additional corrections for multiple comparisons were described. In agreement with the authors, increased relative sigma power during NREM sleep might reflect decreases in relative delta power that reduce sleep depth, which might account for the subjective impression of very light or no sleep among insomniacs who misperceive sleep. Indeed, Krystal et al. [69] also reported significantly lower relative NREM delta power among subjective versus objective insomniacs and versus controls despite no group differences in the time spent in SWS and a negative association between relative delta and the degree of mismatch between subjective and objective measures of sleep after controlling for age, sex, and depression and anxiety scores.

More recently, Spiegelhalder and colleagues [74] recruited 25 primary insomniacs and 29 good-sleeper controls. The sample characteristics and sigma bandwidth (12–16 Hz) were similar to those of Krystal et al. [69]. Spiegelhalder et al. [74] also categorized insomniacs into “subjective” (*n* = 7) and “objective” (*n* = 18) subtypes with criteria similar to Krystal and colleagues [69], except that subjective insomniacs were classified if either (a) TST ≥ 6.5 hr, (b) age < 60 years, TST = 6–6.5 hr, and sleep efficiency > 85%, or (c) age ≥ 60 years, TST 6–6.5 hr, and sleep efficiency > 80%. Given the criteria used, it is noteworthy that the mean age of participants in this study was < 50 years old (Table 1). The study demonstrated significantly higher absolute sigma among the total primary insomnia group during NREM2, but unlike Krystal et al. [69], no differences in sigma activity were found between “subjective” and “objective” insomniacs. Spiegelhalder et al. [74] also reported no statistically significant differences in sigma power during REM sleep. Similar analyses were conducted using other EEG spectral bands, although corrections for potential Type I inflation were not described. Several factors that differentiate this from the earlier study could explain the discrepant findings. Namely, Krystal et al. [69] examined relative sigma during a combined NREM2/NREM3 stage among sleep-maintenance insomniacs, while Spiegelhalder et al. [74] measured absolute sigma during NREM2 and also did not specify recruiting only those with sleep-maintenance insomnia. Spiegelhalder et al. [74] acknowledged the limitations of examining only one sleep recording and restricting primary analyses to NREM2 and REM. However, they noted that an unreported follow-up analysis using overall NREM sleep produced similar results to analyses with only NREM2. Considering the findings of Krystal et al. [69], there may be important differences in (relative) sigma power between subjective and objective insomniac groups during NREM3 sleep that was not captured in Spiegelhalder et al. [74].

A subsequent study by St-Jean et al. [48] reported findings that were inconsistent with Spiegelhalder et al., [74] for differences between insomnia subtypes during NREM and also inconsistent with null findings between insomnia subtypes during REM reported by Krystal et al. [69]. Importantly, criteria used to define subjective and objective insomnia in this study also differed. St-Jean et al. [48] collected four nights of EEG data while the previous two studies each collected only one. As such, PSG-based categorization required the following criteria be met on two consecutive PSG recordings for placement in the subjective insomnia group: (a) TST > 380 min or sleep efficiency ≥ 80% and (b) overestimating SOL by ≥60 min, underestimating TST by ≥60 min, or underestimating sleep efficiency by ≥15% vis-à-vis PSGs and sleep diaries. Individuals with sleep-onset or maintenance difficulties at least 3 nights per week who did not meet these PSG criteria were placed in the objective insomnia group. This study examined absolute and relative sigma power (11–14 Hz) during an overall “NREM” period by manually selecting portions (e.g., >10 min) of each NREM stage (1–4) and of REM from each sleep cycle, excluding periods with miniarousals (0.1–7 sec), microarousals (7.1–14.9 sec), and arousals (>15 sec), movement artefacts, and the 5 minutes preceding and following a stage shift. Results demonstrated that paradoxical (i.e., subjective) insomniacs had higher absolute sigma than primary insomniacs with objective PSG abnormalities during NREM. During REM, subjective insomniacs had lower relative sigma versus controls on frontal and central derivations and lower relative sigma versus objective insomniacs, particularly on F3 and F4. Notedly, subjective insomniacs had greater relative delta (1–4 Hz) versus controls during REM, which could account for the decreased relative sigma power and also suggests that spindle oscillations were inhibited among subjective insomniacs due to increased thalamic hyperpolarization during REM [16]. Finally, a group-by-cycle interaction revealed lower relative sigma among subjective versus objective insomniacs and objective insomniacs versus controls during the 4th NREM-REM sleep cycle on frontal derivations. Overall, St-Jean et al. [48] interpreted their results as indicating how sleep microstructure may be more compromised among individuals with subjective, rather than objective insomnia, in line with the results from NREM sleep reported by Krystal et al. [69]. Lower relative sigma power, during overall REM and in the last sleep cycle, suggests a greater susceptibility to sleep fragmentation among insomniacs during the final hours of sleep. Indeed, St-Jean et al. [48] posited that reduced relative (sigma) power towards the end of sleep time, where REM sleep is typically prominent, could disturb the perception of sleep and account for subjective reports of sleep disturbance in this group. Also, St-Jean et al. reported lower relative sigma during REM in insomniacs but no group differences for absolute sigma, which contrasts with the greater absolute sigma during REM reported by Merica et al. [66]. A noted strength of this study was examining both absolute and relative sigma power during NREM and REM and also from multiple frontal, central, and parietal EEG derivations. Further, while Krystal et al. [69], Spiegelhalder et al. [74], and St-Jean et al. [48] all included criteria for insomnia subtypes based on a minimal TST and sleep efficiency score, St-Jean et al. [48] included criteria for the extent of mismatch between subjective and objective sleep measures (similar to the iterative reanalysis in Krystal et al. [69]) and also required that criteria are met on two (opposed to one) PSG recordings. Such differences could also account for the discrepant between-study results for insomnia subtypes. Other explanations for discrepant findings include the difference in sample sizes among clinical groups and inconsistent periods of sleep chosen for analysis (i.e., NREM2 versus portions of NREM and REM; Table 1). Importantly, St-Jean et al. [48] noted that the nature of their exploratory study precluded correction for Type I error to account for their many analyses, and significance was set at *p* < 0.05; however, post hoc tests for significant group effects and first-order interactions were Bonferonni corrected.

Finally, Cervena and colleagues [77] examined absolute sigma power (12–14.75 Hz) during the 5 minutes before and after the sleep-onset period (defined by the presence of the first visually scored spindle) between good sleepers and individuals with primary insomnia, either with sleep-onset (defined by a PSG NREM2 sleep latency > 20 min on 2 of 3 recording nights) or sleep-maintenance complaints (defined by PSG NREM2 latency < 20 min, wake after sleep onset time > 45 min, and sleep efficiency < 90% on 2 of 3 recording nights). The study found no significant differences in sigma power between groups during the five minutes before or after sleep onset, although differences in sigma power would not be expected prior to initiating sleep. This study's use of objective criteria for insomnia subtypes is a noted strength, but the study is limited by a restricted analysis of the few minutes surrounding sleep onset. Moreover, control data were obtained from only a single recording, while insomniacs all had three recording nights. As such, it is possible that results would have differed if all participants experienced the same 3-night study protocol.

To summarize, studies are generally inconsistent regarding spindle activity differences between insomniacs and good sleepers. It was clear that studies examining a combined or mixed-sample insomnia group typically reported no group differences in spindle activity. One noteworthy exception was Buysse and colleagues [72], who reported a group-by-sex interaction that revealed higher absolute sigma power among female insomniacs. The time-course of sigma power across sleep time does not appear to differ greatly from that of controls, although insomniacs tend to have slower increases/decreases in sigma power across sleep time. Only one study reviewed above [66] reported that insomniacs had significantly lower power curves across the 3rd and 4th NREM cycles but higher power curves across all four REM cycles. However, subsequent studies failed to replicate findings during REM. In contrast to Merica et al. [66], St-Jean et al. [48] reported lower relative sigma power during REM, particularly among subjective (versus objective) insomniacs. Evidence is growing for differences in sigma power between insomnia subtypes. Indeed, differences in spindle activity were more evident among studies that differentiated between insomnia subtypes, although results were still mixed, for example, between selection of the sleep period to be analyzed and between measures in absolute versus relative units (e.g., cf, [48, 69, 74]). Relative sigma power among insomniacs deserves further examination, as decreased relative power in one bandwidth implies greater power in other (i.e., faster or slower) bandwidths; this could help reveal subtle mechanisms underlying the pathophysiology of insomnia. Given the results of Krystal et al. [69], there may be potentially salient differences in sigma power between insomnia subtypes when NREM3 sleep is considered, such as an increase in relative sigma associated with a decrease in relative slow EEG power. However, this remains to be confirmed. Firm conclusions about spindle oscillations in insomnia are difficult to make given the between-study discrepancies in participant selection/recruitment (e.g., sample sizes and criteria for insomnia subtypes) and methodology (e.g., selection of EEG derivations (e.g., F3 versus C3), sleep stage (e.g., NREM2 versus NREM2 + NREM3), spectral bandwidths, and measurement units (absolute versus relative)). The lack of consistent changes across studies might thus well be related to the fact that insomnia is a heterogeneous condition, in which only certain subcategories of individuals would display objective and microstructural changes in sleep architecture. Therefore the characterization of insomnia may require nuanced means of differentiating between subtypes (e.g., based on objective, microstructural (e.g., spindle) differences) beyond broad clinical categories. This approach could offer a more fine-tuned understanding of the functional significance of unique microstructural oscillations between insomnia subtypes and also a way to examine how intervention strategies influence the dynamics of these group-specific EEG parameters. A final noteworthy point is the paucity of studies comparing insomniacs and controls using visual and/or automatic spindle detection methods. While no group differences can be inferred from the study that used event-detection methods [67], group differences were noted among spectral measures (albeit, inconsistently). As such, there is a need for more event-detection studies with both larger and subtyped groups of insomnia patients to help clarify this inconsistency in the literature.

## 4. Hypersomnias/Disorders of Excessive Daytime Sleepiness (EDS)

(1)* Narcolepsy* and* hypersomnia* are classified as disorders of excessive daytime sleepiness (EDS). Cardinal symptoms of narcolepsy include intrusive and overwhelming urges to sleep (sleep attacks) and sleep-onset REM periods (SOREM [63, 94]). Narcolepsy also commonly cooccurs with cataplexy, a temporary loss of muscle tone triggered by emotional stimulation. Pathophysiological studies show that narcolepsy with cataplexy is associated with a central deficit in orexin (hypocretin), a hypothalamic peptide mediating sleep-wake transitions [1] and autoimmune dysfunctions as suggested by the association with HLA-DQB1^∗^0602 [95]. Idiopathic hypersomnia (IH) is characterized by EDS, despite a low sleep debt, and difficulty rising in the morning due to unrefreshing sleep (“sleep drunkenness” [96]), and can be further classified into IH with long sleep time (e.g., >10 hr nocturnal sleep) or without long sleep time. The diagnosis of IH requires careful consideration and exclusion of other potential causes of excessive sleepiness [59, 83]. In contrast to narcolepsy with cataplexy, IH is not associated with SOREM or hypocretin deficiency, and its pathophysiology remains poorly understood. Likewise patients presenting narcolepsy without cataplexy also fail to demonstrate consistent hypocretin deficiency and the mechanisms of narcolepsy without cataplexy also remain unclear.

(i) The literature search identified two studies that examined spindle activity between adults with narcolepsy-cataplexy (NC) and controls and three studies that examined spindle activity between adults with IH and controls (Table 2). Studies of NC will be discussed first. Notedly, both studies only recruited participants with NC, opposed to narcolepsy without cataplexy; one study described in the next (hypersomnia) section also recruited narcoleptics but did not specify if cataplexy was an associated symptom [82]. Between both studies of only NC versus controls, participant ages were largely equivalent, but sample sizes (i.e., *n* = 49 versus 11) and sex proportions were not (Table 2). Both studies examined data from a second recording night using spectral analysis techniques on central EEG leads, but with different sampling rates and analysis epochs. Broadly, observations from this review suggest that spindle oscillations do not differ between NC and controls; however, more research is clearly needed given that only two (inconsistent) studies were retained from the literature search.

Ferri and colleagues [80] examined broad EEG spectrum activity, measured from 0.5 to 25 Hz in 0.5 Hz bins, in 49 NC and 37 controls during each NREM stage and during REM and also among NREM stages scored as various phases of the cyclic alternating pattern (CAP), which reflects patterns of NREM sleep instability. The authors controlled for multiple comparisons with a Bonferroni correction to restrict statistical significance to *p* < 0.0125. Results showed generalized NREM instability among NC versus controls in relation to significant differences in CAP measures, such as a lower percentage of CAP subtype A1 (predominant EEG synchrony and slow rhythms), and higher percentage of CAP subtypes A2 (mixture of slow and fast rhythms, 20–50% EEG desynchrony) and A3 (low-voltage fast rhythms, >50% desynchrony [7]) during NREM sleep. Of note, while NC evidenced a higher percentage of CAP A3 subtype, they also had a lower A3 subtype index (#/hr) versus controls. No group differences were found within the standard absolute or relative sigma band (11–16 Hz) in any sleep stage. However, when examined during CAP phases, NC demonstrated significantly higher power from 5.5–18.5 Hz exclusively in the CAP phase A3 subtype during SWS. Ferri et al. [80] did not interpret this result. Instead, the authors focused their discussion on results from CAP subtype A1 and also hypothesized that reduced orexin levels in NC may account for NREM instability by hindering wakefulness-promoting mechanisms within the thalamus. Overall, they reported evidence for NREM sleep impairments (e.g., increased NREM1 and decreased NREM2 and SWS) in NC that occur alongside REM sleep abnormalities (e.g., short REM-onset latency) and also that faster EEG frequencies associated with REM sleep may leak into NREM periods. However, this requires further study, as the nonspecific increase across faster EEG frequency bands during a CAP phase associated with fast asynchronous EEG could instead reflect microarousal activity resulting from unstable NREM sleep in general, rather than a specific increase in spindle activity, per se. Methodologically, it is worth noting that this study used inconsistent EEG sampling rates (128 Hz or 256 Hz) and derivations to obtain sigma power (C3 or C4) between participants.

Khatami and colleagues [81] recruited 11 NC patients and 11 age- and sex-matched controls. The study examined absolute sigma power (12–15 Hz) during all-night NREM and REM, as well as the temporal distribution (i.e., time-course) of spindle frequency activity (SFA) in a slightly different band (12–14 Hz) across consecutive NREM cycles and also within each NREM cycle. EEG power within 1–20 Hz was examined in 1 Hz bins, but no correction for multiple comparisons was reported; however, the authors focused their discussion largely on results for SFA and SWA. No group differences in absolute sigma power (similar to the previous study) or in relative SFA across NREM cycles were found. However, within-cycle SFA dynamics were impaired in NC relative to controls. Namely, the characteristic U-shape pattern of spindles across the beginning, middle, and end of each NREM cycle was observed only in the first NREM episode in NC but was seen across all cycles in the control group. This abnormal SFA pattern was corroborated by an analysis of correlations between SFA and SWA (0.75–4.5 Hz), which demonstrated that the strength of SFA-SWA associations across NREM episodes was attenuated in NC. However, the discussion largely disregarded findings of spindle activity dynamics between groups and focused instead on attenuated SWA in NC, hypothesized as attributable to greater sleep fragmentation following the first NREM cycle. In line with this, it is possible that an aberrant SFA time-course across NREM cycles reflects a breakdown in thalamocortical mechanisms that regulate slow wave and spindle activity in NC. Given that altered sleep in NC was observed following the 1st NREM cycle, one could also speculate on aberrant mechanisms only revealed as NREM sleep intensity dissipates across sleep cycles. The authors [81] acknowledged that daytime naps taken by those with NC may have impacted the results. Also, given how this study examined a spectral bandwidth that overlaps between slow and fast spindles (12–14 Hz) on C3, where slower spindles are less prominent, it will be important to replicate this study's analyses using a broader sigma bandwidth and distinguishing between both slow and fast spindle activity measured across scalp regions.

To summarize, limited available data suggests that sigma power appears generally unaffected at a group-level between NC and controls. Although results of the first study [80] are largely speculative in regard to spindle activity (but demonstrate NREM instability as per measures of CAP), the second study [81] provided evidence for dysfunctional spindle dynamics among NC, in particular following the first sleep cycle. However, it is important to consider that the second study had a much smaller sample size than the first. Taken together, results of these studies support a generally consistent argument that NC is associated with sleep-wake transition difficulties and problems sustaining consolidated sleep. For example, chronic EDS reported among those with NC might be partially accounted for by poor NREM sleep stability and aberrant SWA/spindle dynamics. This has potential implications for intervention efforts to target NREM instability in NC. Although no differences in spindle activity overall were observed between NC and controls, both studies only used spectral analysis techniques; as such, it would be helpful for studies to examine spindles using event-detection methods and determine if groups differ on measures of spindle density, duration, and amplitude.

(ii) Three studies examining spindle activity between patients with idiopathic hypersomnia (IH) and controls were identified. Only one of these [82] employed a visual event-detection method during NREM2. The other two (more recent) studies both recruited age-matched participants, utilized spectral analysis techniques with equivalent EEG sampling rates, and examined larger portions of sleep. However, between these latter two studies, discrepancies were evident in sample characteristics (sample size (*n* = 10 versus 19); proportion of males (40% versus 68%); age (*M*_age_ = 25.4 versus 46 yrs)) and methodology (number of recording nights (1 versus 2); spectral window duration (4 sec versus 2 sec); sigma bandwidth (12.25–15 Hz versus 11.5–15.5 Hz); see Table 2). Broadly, observations from this review suggest that spindle oscillation differences between adults with IH and controls remain unclear. The first reviewed study suggests greater spindle activity in IH as measured using event-based analysis of spindle density. However, the two studies that examined spectral power were unable to replicate this finding.

Bové and colleagues [82] recruited 11 IH patients, 10 patients with narcolepsy, and 11 normal sleepers. It was not specified whether participants with narcolepsy all had cataplexy. Among the total study group, 56% were male. All participants completed one overnight PSG, and patients also completed a next-day MSLT. Of note, all patients complained of EDS for >1 year before recruitment, but clinical group status was designated based on the absence (IH) or presence (narcolepsy) of a short-onset REM latency during the MSLT. After visually scoring spindles obtained from one recording night, the authors calculated spindle density among individual 10-minute segments of stable NREM2 sleep and derived a mean value for each hemisphere. Between-group comparisons were made by examining an average value of spindle density during NREM2 overall and the temporal distribution (i.e., time-course) of spindle density across each hour of sleep. Both clinical groups combined had significantly greater mean spindle density than controls in both hemispheres, especially at the beginning and end of sleep time. Patients with IH had greater mean spindle density than both other groups, but a statistically significant difference was only reported for all groups together and not between IH versus NC versus controls. However, when examined hour-by-hour, spindle density differed significantly between IH and narcolepsy during the middle of sleep time, where values increased in IH but decreased in narcoleptics (and controls). Further, in contrast to narcoleptics and controls who both showed a tendency towards decreasing spindle density across successive hours of sleep, the IH group instead showed a tendency towards increasing density. The authors discussed whether the increase in spindle density from the middle of sleep time in IH reflected a dysfunctional awakening mechanism and concluded that a high spindle density in IH may signify hyperactivity of NREM sleep oscillation thalamocortical mechanisms which, in turn, interferes with normal waking processes. However, the potential mechanisms underlying this thalamic hyperactivation were not discussed. As well, it remains possible that a first-night effect impacted these results, although the authors supported their decision to examine only one EEG recording by claiming that sleep spindles are unaffected by first-night effects [82].

Subsequently, Sforza and colleagues [83] examined whether participants with IH have either dysfunctional or hyperfunctional sleep homeostatic drive by measuring spectral power in 10 young adult IH patients and 10 age- and sex-matched controls. Specific inclusion criteria for IH in this study (beyond the absence of other sleep disorders/health problems) were severe EDS for >1 yr and mean sleep latency < 8 min on a next-day MSLT without a sleep-onset REM period. However, in contrast to the previous study, an additional criterion was >90% sleep efficiency on overnight PSG. EEG sigma power (and other bands) was measured during NREM sleep and expressed as an overall mean value; the exception was SWA, which was also examined across consecutive NREM-REM cycles. The IH group (versus controls) had lower absolute sigma power (12.25–15 Hz) and SWA power (0.75–4.5 Hz), but only results for SWA reached statistical significance. As the focus of this study was on SWA and sleep homeostasis, results for sigma power between IH and controls were not discussed.

Finally, Pizza and colleagues [84] compared sigma power (11.5–15.5 Hz) between middle-aged IH, NC, and control subjects during successive sleep cycles. Participants completed an initial evaluation that included two overnight PSGs (first: adaptation, second: diagnostic) and a next-day MSLT. Clinical participants were diagnosed based on ICSD-2 criteria and on an MSLT sleep onset latency of <8 minutes. Participants then underwent two additional PSGs for the study (first: adaptation, second: analysis). Pizza et al. [84] examined sigma, along with delta, theta, alpha, and beta power, across the whole night and during portions of NREM2, NREM3, and REM sleep divided into 90-minute periods. Statistical significance was set at *p* < 0.05 across all analyses. In contrast to the pattern observed by Sforza et al. [83], but in line with Bové et al. [82], clinical groups in this study typically showed greater absolute and relative sigma power than controls. However, unlike Bové et al. [82] who demonstrated greater spindle activity in IH than in narcoleptics (who may or may not also had cataplexy), IH patients in Pizza et al. [84] typically fell intermediate to participants with NC and controls. Analyses revealed an overall group effect for absolute sigma during REM sleep in the 3rd 90-minute period, with controls demonstrating less power and NC patients demonstrating more. However, follow-up contrasts between each pair of groups were not performed, leaving it unclear if significant differences existed between each clinical group (IH and NC) versus controls, or only between NC and controls. An overall group effect for relative sigma was also found during whole-night SWS and during SWS and REM specifically in the 2nd 90-minute period, with a similar pattern to that of absolute sigma (NC > IH > Controls). However, as before, follow-up contrasts were not performed between each group pair, and so it remains possible that significant differences in relative sigma were not present between IH and controls. Nevertheless, this result complements Bové et al.'s [82] finding of greater spindle density towards the end of NREM2 sleep time in hypersomnolent patients.

To summarize, three studies of IH were identified, only one of which examined visually scored spindles. The three studies reported inconsistent results for spindle activity between groups, with the first study showing significantly greater spindle density in IH than controls with a narcolepsy group falling intermediate, the second showing no differences in absolute sigma power between IH and controls, and the third showing significantly greater sigma power (relative power, in particular) across different portions of sleep than controls, but with IH maintaining values intermediate to NC and controls. As per Bové and colleagues [82], it is plausible yet unconfirmed if increased thalamocortical spindle oscillations are a mechanism underlying problems with sleep and waking in IH. Given that some evidence suggests a particular dysfunction of increased spindle activity towards the latter half of sleep time, this hypothesis could help explain why patients with IH have such difficulty waking up after a full-night's sleep. Overall, this review identifies a gap in the literature regarding spindle activity in IH versus controls. Further, none of the studies examined spindle oscillations in frontal or parietal derivations, and none examined slow versus fast spindle oscillations. Finally, no study reviewed in this section so far has specifically recruited narcolepsy patients without cataplexy; indeed, a systematic examination of spindle activity between narcoleptics with and without cataplexy and IH is especially lacking.

(2)* Sleep-disordered breathing* (*SDB*) is a broad category used to describe interruptions in the normal rhythmicity, depth, and regulation of breathing during sleep. SDB is categorized under obstructive events, where apneas occur with continued or increased inspiratory effort (snoring, obstructive sleep apnea/hypopnea; OSAH), central events, where apneas occur without coinciding inspiratory effort (central sleep apnea; CSA), and mixed events, where inspiratory effort gradually increases across the minimum 10 sec event duration [8]. The apnea-hypopnea index (AHI), or the number of apneas/hypopneas per hour of sleep, is often used as an objective measure of disease severity. Standard AHI cut-off scores remain to be established, although values ≥15 are often considered indicative of moderate or severe OSA [97]. A similar measure, the respiratory disturbance index (RDI), includes apneas, hypopneas, and other respiratory events not scored as apneas or hypopneas but that still disturb sleep. Respiratory events can be modulated by sleep stage and sleeping position and typically cause arousals that disturb both sleep continuity and depth. Risk factors, comorbidities, and health consequences of SDB have been extensively researched. Primary risk factors include heavy smoking/alcohol/sedative-hypnotic drug use, snoring, male sex, obesity, and older age [98, 99]; particularly in elderly populations, those at risk for SDB would likely also show declines in multiple cardiovascular, pulmonary, and muscular functions [100]. Overall, SDB places individuals at increased risk for morbidity and mortality [98].

(i) Nine studies that examined spindle activity between participants with SDB and controls were identified (Table 2). Sample sizes were typically 15 or less, with one study [91] recruiting 27 clinical participants. Clinical participants were generally middle-aged, and sex distributions among studies were approximately even across patient samples; two noteworthy exceptions include one study with an all-male sample [89] and another [90] with a mostly male sample (93%). Reviewed studies focused exclusively on patients with obstructive (instead of central) respiratory events. With only a few exceptions, most patients were recruited into a mixed/combined clinical group with a range of disease severity (mild, moderate, and severe). Methodologically, 4 studies [85, 86, 88, 91] used only manual and/or automatic spindle detection methods, and the remainder used spectral analysis techniques. However, Ondze et al. [87] examined both spectral sigma power and an hourly sleep spindle index (SSI) derived from an automatic sleep analyzer. All studies of spectral power analyzed only one night of EEG data, except for Ondze et al. [87] who examined data obtained following an adaptation. Further discrepancies include EEG sampling rates and sigma bandwidth definitions. Finally, while most studies focused on longer sleep periods (e.g., NREM2; NREM-REM cycles), some examined EEG power changes around respiratory cycles or respiratory events; the two types of analyses will be considered separately. Broadly, observations from this review suggest that adults with SDB tend to have fewer and slower spindle oscillations versus controls.

Among OSA studies employing an event-detection analysis of spindles, the earliest was by Himanen and colleagues [85]. Inclusion criteria for OSA patients were subjective complaints consistent with ICSD criteria (e.g., daytime sleepiness or headache and/or dry mouth upon waking), and an AHI > 10/hr; the median AHI among patients was 23 (range = 10–50). Spindles were visually scored by two independent raters, each examining only one hemisphere; only synchronous bilateral spindles, ones that occurred at the same point in time on each hemisphere, were accepted for analysis. Interscorer agreement of spindles between left and right hemispheres was reported as adequate at 80%. Spindles were examined across consecutive NREM episodes, with each episode also divided into 10 equal intervals. Results first showed a nonsignificant trend for reduced median spindle count and density among the OSA group versus controls. Next, the authors examined spindle frequencies across EEG derivations using spectral analysis methods and reported that detected spindles were typically slower in OSA (11.9 Hz) versus controls (12.9 Hz). Significant between-group differences in spindle frequency (i.e., slower spindles) were observed among many of the individual intervals within a NREM episode, mostly at the beginning and end of each NREM episode; the greatest number of significant differences was found in the 3rd and 5th NREM episode and the fewest number of differences in the first two episodes. In the same year, Huupponen and colleagues [86] compared spindles between OSA and controls using an automatic spindle detector on data derived from a preliminary FFT algorithm. As with the previous study, inclusion criteria for patients were subjective complaints consistent with ICSD criteria for OSA and an AHI > 10/hr; 70% of patients had an AHI ranging within 22–50/hr, and 30% had an AHI < 20/hr, suggesting a mixed-sample of disease severity. Huupponen et al. [86] examined the total number of spindles and spindle frequency but unlike Himanen et al. [85] did not report spindle density. Results were highly similar with the visually scored spindles in [85]: while no group differences were found in the number of spindles, patients had significantly slower spindle frequencies than controls (11.7 Hz versus 12.5 Hz), particularly during the 3rd, 4th, and 5th NREM cycles. Both Himanen et al. [85] and Huupponen et al. [86] concluded that OSA patients have a general abundance of slower spindles throughout the night versus controls, and Himanen et al. hypothesized that this reflects left-over homeostatic pressure caused by frequent arousals and sleep instability.

Ondze and colleagues [87] examined spindle activity using a measure of the sleep spindle index (SSI) derived via an automated sleep analyzer that employed integrated digital filtering of 13–17 Hz activity. Patients were middle-aged adults with mild SDB, defined by the presence of snoring, sleepiness/daytime fatigue, a respiratory disturbance index (RDI) between 5 and 30/hr, and a periodic limb movement index < 5/hr. In contrast to the previous two studies, results showed multiple group differences in the number of detected spindles across several periods of sleep. Namely, the SSI was significantly lower in the mild SDB group during total overall (i.e., whole-night) sleep, within each of 4 consecutive NREM-REM sleep cycles, during each of NREM2 and NREM3 sleep when examined overall across the night, and during each of NREM2 and NREM3 when examined across consecutive sleep cycles. In a similar vein to Himanen et al. [85], Ondze and colleagues posited that reduced spindle activity among patients has less to do with an increase in SWA and instead more likely reflects greater sleep fragmentation; indeed, this group of patients had significantly more arousals versus controls in all sleep stages except REM.

A more recent study by Carvalho and colleagues [88] examined spindles (11–16 Hz) in patients with OSA grouped based on their AHI: mild OSA (5–14/hr; *n* = 11) and moderate OSA (15–29/hr; *n* = 10), compared to 7 healthy controls. The authors used a unique approach to spindle analysis by quantifying within-spindle frequency variability, referred to as “chirp,” using an automatic spindle detection algorithm. Chirp accelerations and decelerations reflect a given spindle's tendency to increase (“positive” chirp; >0 Hz/sec) and decrease (“negative” chirp; <0 Hz/sec) in frequency across time, respectively, with negative chirping (i.e., spindle frequency decelerations) demonstrated as more common than positive chirping [101]. Carvalho et al. [88] examined 30 minutes of NREM2 (10 mins from the initial, middle, and final portions of sleep). Spindles were also divided into slow (<13 Hz) and fast oscillations (≥13 Hz). Three primary findings in this study are worth reporting here. First, the percentage of negative chirp among slow spindles was significantly lower in the moderate OSA group versus mild OSA and controls on frontal and parietal regions, but no differences were found between mild OSA and controls. Second, there was a decreasing frontoparietal gradient in negative chirp percentage among slow spindles that reached significance only within the moderate OSA group, but not others. Third, the overall median chirp rate (i.e., amount of change in Hz/sec) demonstrated a loss of spindle deceleration among slow spindles in moderate OSA (Med (SD) = 0 (0.67) Hz/sec) versus mild OSA (−0.11 (0.7) Hz/sec) and controls (−0.08 (0.6) Hz/sec), but only on parietal derivations. Similar results across each of the three analyses were not obtained when only fast spindles were considered. Carvalho et al. [88] suggested that across-time changes in the frequency of individual spindles are modulated by internal spindle-generation mechanisms and also that reduced chirp rates among parietal slow spindles may reflect a unique cortical response to arousals and sleep instability in moderate OSA. Building on this, it is also possible that decelerations in the frequency of parietal slow spindles become progressively slower with greater disease severity.

Among SDB studies that employed spectral analysis of absolute spindle activity, results were somewhat discrepant between Guilleminault et al. [89] and Ondze et al. [87] (described above), although both utilized the same sigma bandwidth (12.25–15 Hz), examined overall, and measured across individual 1 Hz or 0.25 Hz bins (resp.), and both reported abnormally higher delta power towards the end of the night. Guilleminault et al. [89] recruited 36 overweight, but nonobese, age-, sex-, and BMI-matched adults with sleep-disordered breathing, divided into 3 clinical groups based on a preliminary diagnostic PSG: upper airway resistance syndrome (≥10 respiratory events + arousals per hr that do not meet criteria for an apnea or hypopnea; AHI = 1.63/hr); obstructive sleep apnea (≥80% of respiratory events meeting criteria for apnea; AHI = 34.2/hr); and obstructive sleep hypopnea (≥80% of respiratory events meeting criteria for hypopnea; AHI = 36.2/hr). Results showed lower sigma power specifically around the 13-14 Hz band in all clinical groups versus controls during NREM, but higher sigma power during total (NREM + REM) sleep, and no difference during REM. Of note, results for sigma power differences between individual patient groups were not reported, leaving it unclear if one patient group had a greater difference in sigma power versus another patient group or versus controls. Divergent with Guilleminault et al. [89], Ondze et al. [87] reported significantly lower (opposed to higher) absolute sigma during total whole-night sleep and lower sigma during each of four consecutive NREM-REM sleep cycles among mild SDB versus controls. Both authors interpreted their results in relation to general hyperarousal, sleep fragmentation, and aberrant homeostatic sleep pressure dynamics in sleep-related breathing disorders. As well, both studies examined multiple spectral variables across NREM, REM, and whole-night sleep, but neither reported accounting for potentially inflated chances of Type I error.

Of the remaining studies examining relative sigma power in OSA, each had largely similar sample sizes, EEG methods, and EEG sigma bands. As such, results of these studies can be compared with greater ease (see Table 2). Abdullah and colleagues [93] demonstrated significantly lower relative sigma power (13–16.5 Hz) among a mixed/combined group of OSA patients (mean (SD) AHI = 48.97 (27.52)/hr) versus controls during NREM2 (*p* = 0.005); patients also had significantly lower relative theta (5–8.5 Hz) and beta power (17–30 Hz) during NREM2, but no group differences were reported for delta or alpha in this sleep stage, or for sigma power in other sleep stages. The authors did not interpret results for sigma power, specifically. However, when considered alongside lower relative beta power and a higher sleep efficiency versus controls (87.9 versus 82.7%; significance not reported), lower relative sigma in OSA could suggest this group maintained a greater potential for arousability than controls, even if they did not wake up more often across the night. Conversely, Dingli et al. [90] found no differences in relative sigma power (12–16 Hz) between a mixed group of 15 OSA patients (mean AHI = 29/hr, range = 10–79/hr) and 7 controls during NREM, REM, or total sleep, and Xavier et al. [92] reported only a nonsignificant decrease in sigma power (12–16 Hz) among OSA versus controls during total sleep. Of note, Xavier et al. [92] only reported that patients had been diagnosed with OSA and did not report disease severity or provide other relevant details (e.g., mean AHI). Considering the three studies all together, there may be differences in relative sigma power between OSA and controls during NREM2 that were not captured when only overall NREM sleep or total/whole-night sleep are considered.

Finally, some studies of SDB also investigated sigma power changes chiefly during respiratory cycles. Chervin et al. [91] examined the degree to which EEG spectral activity, derived from digital filtering, varied in synchrony with respiration among normal (i.e., nonapneic) respiratory cycles between 27 SDB patients with mixed diagnoses (*M* (SD) AHI 25 (21.8)/hr) and 11 controls. Respiratory-cycle related EEG changes were measured using relative EEG power, calculated by dividing the power in a given bandwidth for each of 4 respiratory cycle segments (early/late expiration, early/late inspiration) by the power in that bandwidth during the entire (nonsegmented) respiratory cycle and then averaging across all corresponding segments within the first 3 hours of sleep. No adjustments for multiple comparisons were discussed, and the common 0.05 level of statistical significance was applied for all analyses. Results showed no group differences in respiratory cycle-related changes in sigma power (13–15 Hz), or other bands, versus controls, although significant within-group variations were observed. Chiefly, sigma power in both groups showed an initial decrease from the 1st to 2nd respiratory segment and an increase from the 3rd to 4th respiratory segment; however, the extent of relative sigma power remained stable from the 2nd to 3rd segment among SDB participants but increased from the 2nd to 3rd segment in controls. Similar within-group variations were observed in both groups for relative alpha power, while a significant within-group variation in relative delta power was observed only among controls. As well, using regression models, the authors demonstrated that the degree of change in sigma power across the four respiratory cycle segments significantly predicted next-day sleepiness in SDB. Overall, the authors suggested that sleep disruptions (e.g., altered EEG power) among SDB may not be restricted only to apnea/hypopnea events. Two additional studies examined EEG power specifically around respiratory events during sleep. Dingli et al. [90] observed a significant elevation in sigma power among OSA patients in the 10 seconds following individual respiratory events associated with an arousal (versus without an arousal) during NREM and total sleep, compared to the 10 seconds before. However, this result was minimally discussed, as the authors focused more on theta power (significantly lower in OSA, attributed in part to greater relative delta and sigma power). Dingli et al. [90] speculated that EMG elevations coinciding with an arousal could have contaminated the EEG signal and distorted measures of alpha, sigma, and delta. While this is possible, a genuine increase in sigma power following respiratory events (and especially events with arousals) might reflect the sleep maintenance hypothesis of spindle activity. In contrast to Dingli et al. [90], Xavier and colleagues [92] examined EEG power changes between the 10 seconds before and after a respiratory event and demonstrated that OSA patients had only nonsignificant reductions in sigma power following respiratory events. Xavier and colleagues [92] suggest that their decision to not separate between sleep stages accounts for the discrepant findings between their study and that of Dingli et al. [90]. Although it was not discussed, another difference was that Xavier et al. [92] did not differentiate respiratory events with and without arousals.

To summarize, studies were somewhat varied in regard to spindle/sigma activity between OSA and controls. However, a relatively consistent finding was that OSA patients tended towards less spindle activity and slower spindle frequencies. Event-detection studies demonstrated that OSA patients have an abundance of slower spindles throughout the night, with one other study demonstrating significantly lower spindle counts in mild SDB. Findings of altered spindle characteristics in SDB were advanced by Carvalho et al. [88], who demonstrated that the moderate OSA group had a decreased rate of deceleration across time (“chirp”) among parietal slow spindles versus mild OSA and controls. Among spectral analysis studies, one study reported lower absolute sigma power in SDB versus controls during NREM, but higher sigma during total/whole-night sleep, while the second study reported lower absolute sigma in SDB during both total sleep and across each NREM-REM sleep cycle. Only one of three studies examining relative sigma demonstrated lower power in OSA, specifically during NREM2 [93]. Studies of respiratory-related changes in sigma power provided largely inconsistent results, likely due to stark between-study differences in methodology. Decreased spindle activity in SDB could reflect aberrant SWA dynamics or increased sleep fragmentation, but this is a distinction that requires further testing. However, given that fewer spindles suggest greater arousability, a potential “consequence” of attenuated spindle activity in SDB is that cortical arousals can occur with greater ease in the face of potential asphyxiation during sleep. Importantly, given that most of the reviewed studies examined a combined group of SDB patients with mixed disease severity, it is possible that differences between groups would become more evident with more careful differentiation between mild, moderate, and severe SDB subtypes. Also, while there was an array of studies with OSA that examined detected and spectrally analyzed spindle activity, only one article used both methods in the same study [87]. As such, future researchers should consider examining both detected spindles and spectral sigma power in the same study to help further elucidate differences in spindle activity between groups. Given that most reviewed studies examined only central EEG derivations (see Table 2), a topographical analysis might be particularly useful in examining across-scalp differences between fast and slow spindles in SDB.

## 5. Sleep-Related Movement Disorders/Parasomnias

(1)* Restless legs syndrome* (*RLS*) is a sleep-related movement disorder characterized by profound urges to move one's legs during periods of rest or sleep, which provides temporary relief of recurring pain and discomfort, and is associated with impaired sleep, daytime dysfunction and fatigue, and reduced quality of life and relationships (e.g., difficult for bed partners; [59, 61]).* Periodic limb movements in sleep* (*PLMS*) is another movement disorder characterized by repetitive limb movements (e.g., toe extension), typically during NREM sleep, which last 0.5–10 sec and require a minimum of 8 *μ*V increase in EMG voltage from baseline [8]. PLMS rarely cause full arousals from sleep but can impair sleep depth and continuity, although some individuals with PLMS may be unaware of the sleep disruption and feel asymptomatic [59, 61, 96]. Of note, RLS is often accompanied by PLMS, but the opposite is not typically observed. As such, it is important to remember the salient distinctions that exist between RLS and PLMS. Both conditions can be triggered and exacerbated by the effects of multiple pharmacological agents (see [102] for a review). RLS and PLMS are common in older men (>50–60 yrs) and pregnant women and are comorbid both with each other and with other sleep (e.g., insomnia and narcolepsy) and psychological disorders (e.g., depression and anxiety [61, 94, 103]). RLS is associated with increased risk for cardiovascular morbidity [104].

(i) Two studies that examined spindle activity between participants with RLS and controls were identified. Neither study examined participants with PLMS exclusively (Table 3). Samples included middle-aged adults with approximately even sex distributions, but generally small sample sizes, with RLS group sizes of *n* = 20 and 27. In both studies, participants were free of sleep-altering medication at least for a predefined washout period prior to sleep recording, and data were obtained using spectral analysis. Compared to Hornyak et al. [105], the more recent study by Ferri et al. [106] obtained two nights of data using a slower EEG sampling rate and narrower sigma band and focused their analyses around the 5 minutes before and after sleep onset (described further below). Broadly, observations from this review suggest that spindle oscillations do not differ between adults with RLS and good-sleeper controls. However, more research is needed given the methodological inconsistencies between the two studies retained from the literature search.

Hornyak and colleagues [105] examined absolute sigma power (12–16 Hz), and other EEG bands, during whole-night NREM and REM sleep (either including or excluding epochs with arousals or leg movements), and also during epochs selected from NREM2 and REM sleep that contained (a) leg movements without an arousal, (b) an arousal without a leg movement, and (c) a leg movement with an associated arousal. Patients (*n* = 20) were diagnosed in accordance with international criteria for RLS (i.e., urge to move the legs to relieve unpleasant sensations, worsening of urges to move during periods of rest/inactivity, partial or total relief of discomfort after moving the legs, and worsening of symptoms in the evening). All participants were free of medication for at least 2 weeks prior to study, although it was noted that some RLS patients took other medications such as beta-blockers and estrogen, which could have still impacted study results. Interestingly, while the authors also acknowledged the potential of a “first night effect,” they noted how using a single recording night was intended to foster movement-related arousals that would not be expected if participants were acclimated to the laboratory. Indeed, RLS patients had significantly more leg movements and arousals versus controls. While the authors established statistical significance at *p* < 0.05, they reported accounting for Type I inflation by using MANOVAs and, with a significant result, follow-up ANCOVAs to check for significant univariate main effects. The study demonstrated no between-group differences in sigma power during whole-night NREM or REM sleep, both when arousals and leg movements were included or excluded, and no between-group differences in the secondary analysis of individual epochs with arousals and/or leg movements during NREM2 and REM. However, significant within-group effects were observed in both RLS and controls, with absolute sigma increasing from baseline to epochs containing leg movements with associated arousals during both NREM2 and REM. Based on the lack of between-group differences across most EEG bands, Hornyak et al. [105] suggested that the effects of leg movements on EEG power are minimal and might not account for reduced sleep quality and continuity in RLS; instead, they assert that RLS may be characterized more by a motor-related pathology opposed to EEG dysfunction or nocturnal hyperarousal. Future researchers should consider replicating this study using relative sigma power and also including an additional comparison group with frequent PLMS but who are not diagnosed with RLS.

Almost a decade later, Ferri and colleagues [106] examined absolute and relative sigma power (11.75–14.75 Hz) in the ten minutes surrounding the sleep onset period (SOP), defined by the occurrence of the first sleep spindle. The authors examined EEG power during the five minutes preceding (SOP1) and following sleep onset (SOP2) in 27 RLS patients, who also complained of sleep-onset insomnia, and compared this group with 11 sleep onset/maintenance insomniacs, and 14 good sleepers; participants had comparable demographics to participants in [105], but data were analyzed from a second recording night and collected with a slower sampling rate (128 versus 256 Hz). Similar analyses were conducted across other standard EEG bands. While Ferri et al. [106] demonstrated that absolute sigma power was significantly elevated during SOP1 in RLS versus controls, and in insomniacs versus controls, this finding is difficult to relate to spindles given that EEG power in the traditional sigma band prior to sleep onset (which, by the author's definition, was indicated by the first sleep spindle) more likely reflects wake or NREM1 brain activity opposed to spindle oscillations. No group differences between RLS and controls were reported for absolute sigma power during SOP2, or for relative power during SOP1 or SOP2. As well, no significant differences in absolute or relative sigma during SOP1 or SOP2 were reported between RLS and insomniacs, although a trend was observed for higher sigma power among insomniacs. Ferri et al. [106] did not interpret findings for sigma power between groups. Instead, their discussion of EEG power focused largely on greater alpha and beta power in both clinical groups versus controls, and they reported on finding support for a hypothesis that both RLS and insomnia patients display hyperarousal during the sleep onset period. Overall, this interpretation contrasts with Hornyak et al.'s [105] assertion that RLS may be unrelated to nocturnal hyperarousal. However, the divergent interpretations may be attributed to how Ferri et al. [106] examined only the minutes surrounding sleep onset, while Hornyak et al. [105] examined larger portions of NREM and REM sleep with or without leg movements and/or arousals.

To summarize, the two reviewed studies of spindle oscillations in RLS both found no significant group differences versus controls during different periods of sleep. Although caution is warranted given the methodological differences (i.e., NREM and REM sleep with/without leg movements and arousals versus the 10 minutes surrounding sleep onset), results tentatively suggest that sigma power in RLS may not deviate greatly from controls. As such (and consistent with [105]), instead of poor sleep in RLS resulting from attenuated EEG (sigma) power during sleep, it remains possible that poor sleep results from other factors that modulate leg movements themselves and the expression of subsequent cortical arousals. Thus, mechanisms underlying sleep disruption in RLS might be more readily observed at a subcortical rather than cortical level, but this would require empirical testing with specialized tools such as subcortical/depth EEG or functional brain imaging equipment. Overall, there is a paucity of research examining sigma activity between RLS and control groups. It is also important to note that neither reviewed study of RLS examined spindles from visual/automatic detection analyses, making this a pertinent area for future study.

(2)* Parasomnias* are abnormal physiological, psychological, and/or behavioural events that occur during NREM (e.g., sleepwalking and sleep terrors) or REM sleep (e.g., nightmare disorder and REM sleep behaviour disorder (RBD)), or during sleep-wake transitions (see [113] for a review). Parasomnia (PA) episodes can be disturbing (if not frightening) or dangerous and often involve behaviours that might appear goal-oriented but are largely automatic or unconscious and infrequently recalled upon waking [61]. Sleepwalking, or somnambulism, can include simple (e.g., arm rising) or more complex behaviours (e.g., leaving the bed and opening doors). Sleep terrors involve waking in a state of fear (e.g., shouting and thrashing) and physiological hyperarousal (e.g., increased heart rate and sweating). Both sleepwalking and sleep terrors are often considered as “disorders of arousal” that typically occur during SWS in the first third of the night and often cause unresponsiveness and/or confusion in the affected person [63, 94]; moreover, both experiences can be triggered by drug effects, sleep deprivation, alterations in respiratory and hormonal activities, and stress [56]. Nightmare disorder involves recurrent, emotionally charged, and often frightening dream experiences that often occur during later REM periods; nightmares coincide with fear, anxiety, and physiological hyperarousal upon waking and occasional apprehension about returning to sleep [61]. Differentiating features between night terrors and nightmares are their timing (earlier versus later), level of arousal (partial versus full), and extent of postevent recall (less versus more; [94]). RBD is primarily characterized by a loss of muscle atonia and excessive muscle activity during REM [8]. The disorder can result in violent and often uncoordinated behavioural enactments to presumably frightening dreams and is associated with cognitive impairment [114] and a higher incidence of neurodegenerative disorders (e.g., Parkinson's disease, multisystem atrophy [115]). Sleepwalking, nightmares, and sleep terrors are more common in children than adults, whereas RBD is chiefly seen in older adults (>50 yrs [61]), especially in older men.

(i) Six studies that examined spindles between participants with a PA and controls were identified (Table 3). More specifically, two studies recruited participants with sleepwalking and/or night terrors, one recruited participants with chronic nightmares, and three studies recruited participants with RBD. The two studies of NREM parasomnias will be reviewed first, and remaining studies of REM sleep parasomnias will be reviewed second.

Among the two studies examining adults with sleepwalking and/or sleep terrors, samples sizes were small (*n* = 10 or 11), and participants were typically younger than in studies with other sleep disorders. While both studies compared data from clinical samples with that of age- and sex-matched controls, Espa et al. [107] had a lower proportion of male participants than Guilleminault et al. [108] (see Table 3). Espa et al. [107] used both an automatic spindle detector and a measure of spectral EEG power on a second recording night, while Guilleminault et al. [108] only utilized spectral analysis with data obtained from one recording. Other methodological differences across these studies are noteworthy and include differences in the EEG derivations analyzed for spectral power, the speed of EEG sampling rates, and the definitions of sigma frequency bandwidths (Table 3). However, both studies examined relatively large periods of NREM sleep and analyzed spectral power using similar mini epoch durations (4 sec epochs). Broadly, observations from this review suggest that spindle oscillations between adults with PA and good-sleeper controls may be evident when examined using event-detection analyses, while fewer differences are detected with spectral analysis of sigma power. However, more research is needed given the methodological inconsistencies between the two studies retained from the literature search.

Espa and colleagues [107] recruited 11 participants with sleepwalking and/or sleep terrors (with onset at/before 15 yrs), selected based on subjective complaints of sleepwalking or sleep terrors several times per month, a history of PA verified by a third party, and a clinical interview. Following a standardized 16-hour wake period, the authors examined absolute spectral power during overall NREM and REM sleep, a measure of relative power during the 2nd, 3rd, and 4th NREM and REM cycles expressed as a percentage of power in the 1st NREM and REM cycle, and an hourly sleep spindle index (SSI; 13–17 Hz) derived from an automated sleep analyzer that used integrated digital filtering. No between-group differences were reported for spectral sigma power during NREM or REM sleep. However, significant differences were noted for the SSI. Namely, the PA group had a significantly decreased SSI compared to controls overall and decreased SSI during the 2nd and 4th NREM-REM sleep cycles. When examined more closely, the PA group had a specific reduction in SSI during total (whole-night) NREM3 sleep and during the 2nd and 4th NREM3 sleep cycle. As well, control subjects evidenced a gradual increase in SSI across consecutive NREM-REM sleep cycles during the night (with significant increases in SSI from one cycle to the next), but the same pattern was not seen among PA, who instead showed a significant decrease in SSI from the 1st to the 2nd NREM-REM cycle, followed by a significant increase between the 2nd and 3rd cycle, and a nonsignificant decrease from the 3rd to the 4th cycle. A primary focus of this study was on SWA, which, in PA versus controls, was distributed more evenly throughout the night, decayed more slowly across time, and was higher towards the end of sleep. Espa and colleagues [107] interpreted an abnormal SWA distribution in PA as reflecting recurrent awakenings from NREM3 that interrupted the normal distribution of SWA observed in controls and posited that NREM parasomnia episodes require both increased slow-wave-sleep pressure and a higher occurrence of arousals; indeed, PA subjects had greater indices of arousal and sleep fragmentation mainly during NREM3. Espa et al. [107] related results of the SSI analyses (lower in PA in NREM-REM cycles 2 and 4 and during NREM3) to aberrant SWA dynamics by suggesting that a sustained presence of slow waves inhibited the generation of spindle activity in PA versus controls. This study has several strengths, including a standardized recording protocol and examining both shorter and longer periods of NREM and REM sleep. However, while heterogeneity among research participants with a PA such as sleepwalking is difficult to avoid, a limitation not discussed by the authors was that measures of EEG power may have been biased by recruiting adults who have sleepwalking and/or night terrors, with PA episodes occurring at a self-reported frequency of 1/night to 4/month.

Guilleminault and colleagues [108] compared several spectral bands between 10 sleepwalkers (without other PAs) and 10 age- and sex-matched controls that were randomly selected from a larger prospective study examining treatments for nonoverweight sleepwalkers. Analyses of relative power were conducted on EEG data obtained from a one-year follow-up recording, after isolated treatment of an underlying mild SDB (present in all patients) helped reduce the occurrence of sleepwalking episodes. The authors described examining relative EEG power; however results were reported in micro volt-squared (i.e., *μ*V^2^, absolute) units instead of in a percentage or proportion metric. The authors note that post hoc contrasts following one-way ANOVAs were adjusted with a Bonferroni correction. No significant differences in relative sigma power (12.25–16 Hz) were found between sleepwalkers and controls, although the mean power in sleepwalkers was less than half the value obtained from controls (31.7 versus 76.0 *μ*V^2^). Several noteworthy differences can be identified between this and the previous study. Namely, Guilleminault et al. [108] examined relative sigma power during a nonsleepwalking night in young-adult sleepwalkers who had an underlying mild SDB, while Espa et al. [107] examined absolute sigma power during a recording night that contained PA episodes in a group of participants who exhibit sleepwalking and/or sleep terrors. These differences, in addition to those outlined above, make it challenging to draw a meaningful comparison between the two studies.

To summarize, both studies of NREM parasomnia reported no group differences in sigma power versus controls, although sample (e.g., diagnostic group) and methodological differences (e.g., frequency band selection) were readily observed. However, Espa et al. [107] demonstrated significantly lower SSI in PA during the 2nd and 4th NREM-REM cycle and during NREM3 in particular. While further testing is required, this result suggests a problem with the normal gating of cortical arousals during NREM3, which could help explain the occurrence of PA episodes during transitional states around periods of deeper sleep. More research using both event-detection and spectral analysis methods, especially with larger samples, is certainly required to understand the role of spindle activity in PAs.

(ii) Three studies examined spindles in adults with RBD and one study examined spectral sigma power in young adults who suffer from chronic nightmares. Among studies of RBD, sample sizes ranged from 15 to 35 participants, but sample ages were more equivalent (*M*_age_ range = 60.1–66.6 yrs; Table 3). Demographic characteristics like these are expected among studies of RBD. While two studies recruited only patients with RBD and healthy controls, Christensen and colleagues [109] also recruited age-matched participants with (a) RBD and Parkinson's disease, and (b) Parkinson's disease only. Moreover, the studies by Christensen et al. [109] and O'Reilly et al. [110] both used automatic spindle detection methods while Massicotte-Marquez and colleagues [111] utilized spectral analysis. While two studies used a sampling rate of 256 Hz, one study [109] failed to report their EEG sampling rate. Finally, the studies by Christensen et al. [109] and Massicotte-Marquez et al. [111] examined only one night of PSG data (i.e., no adaptation night), while O'Reilly et al. [110] did not report this detail. Broadly, observations from this review suggest that adults with RBD have reduced spindle density during NREM sleep versus healthy controls, but differences may be less evident among measures of spectral sigma power, as demonstrated by the third reviewed study. The one study examining spindles between chronic nightmare sufferers and controls did not yield significant group differences.

Christensen and colleagues [109] recruited four groups of older middle-aged participants (*n* = 15 each): idiopathic RBD (iRBD), Parkinson's disease + RBD (PD + RBD), Parkinson's disease without RBD (PD − RBD), and healthy controls. The density of sleep spindles (11–16 Hz) was detected using both manual and automatic scoring on F3 and C3, following a single night recording during both NREM (total and individual stages) and REM. The spindle detector developed in this study used preliminary filtering and amplitude detection and was followed by a Matching Pursuit method and an automated algorithm applied to a training data set for classification of spindles. A validation check was performed between automatically and manually detected spindles and the authors reported that the algorithm's sensitivity (84.7%) and specificity (84.5%) were both adequate. While there were no between-group differences in spindle density during NREM1, the iRBD and PD + RBD groups both had significantly lower spindle densities versus controls during NREM2, NREM3, and total NREM sleep at a significance level of 0.01; when the significance threshold was reduced to 0.05, the PD group also had a lower spindle density than controls during NREM2 and total NREM. No differences were obtained during REM. This study only provided results for spindles between each clinical group versus controls and did statistically compare data only between iRBD, PD + RBD, and PD − RBD. However, the pattern of spindle density between each clinical group during NREM2, NREM3, and total NREM sleep indicated that PD + RBD had the fewest spindles and that iRBD fell intermediate to each PD group. The authors interpreted their findings by suggesting that progressive neurodegeneration seen across normal aging, iRBD, PD, and finally PD + RBD, might lead to a greater degree of damage to thalamocortical spindle generators, thus leading to the suggestion that spindle density can act as a biomarker for PD. While a strength of this study is the comparison between 3 clinical groups and healthy controls, clinical participants with PD were not required to discontinue dopaminergic pharmacotherapy for this study; the authors only noted how a dose-response effect could not be tested and that they do not believe this would have impacted spindle activity. As well, the authors did not report whether clinical participants were selected based on the absence of other sleep disorders, stating only that this was a criterion enforced for controls. Finally, small but noticeable differences across all four groups were evident for mean age and body mass index, with controls as younger and weighing less and PD + RBD as older and weighing more; however, the authors did not conduct statistical tests to examine whether demographic differences existed between groups.

O'Reilly and colleagues [110] used an automatic algorithm to detect spindles (11–16 Hz) among 35 patients with RBD and 35 healthy controls. Importantly, while diagnosis was verified by a physician via clinical history and a PSG examination, the authors did not explicitly state if only patients with idiopathic RBD (versus secondary or drug-induced RBD) were recruited. As well, compared to the spindle detection algorithm used by Christensen et al. [109], the detector used here was based on an algorithm that band-pass filtered raw EEG signals in the spindle frequency range in both a forwards (starting at the beginning of a recorded segment) and backwards direction (starting at the end) and then determined if the root-mean-squared amplitude (calculated using 0.2-sec overlapping windows) fulfilled both amplitude (≥92nd percentile) and duration (0.5–2 sec) threshold criteria. Data from 19 EEG electrodes (with different sample sizes available from each derivation) were pooled from various studies and clinical assessments across a 10-year period. The authors proposed a topographical examination of multiple spindle parameters during NREM2, including mean density, duration, root mean square amplitude, frequency slope, and mean frequency; however, because no significant differences were obtained for other parameters during a preliminary screening, analyses were then restricted to an examination of only mean spindle density. The authors also examined data derived using individualized thresholds for slow versus fast spindles opposed to a standard amplitude for all participants but noted that results were similar when fixed thresholds were applied. Results demonstrated that, despite no differences in sleep macrostructure, overall spindle densities of RBD patients were 6–35% lower, depending on scalp region, versus controls. In particular, lower fast spindle densities in patients were observed across all EEG derivations except Fp1 and Fp2, but higher slow spindle densities in patients were observed across frontal (F3, F4), central (C3, C4), parietal (P3, P4), occipital (O1, O2), and temporal derivations (T3, T5, T6). O'Reilly et al. [110] hypothesized that attenuated fast spindle densities in RBD reflects a risk for learning and memory deficits associated with neurodegeneration and proposed that longitudinal examinations of spindle activity in RBD are a useful future direction to help clarify across-time relations between cognitive decline and spindle oscillations in this group. The authors did not provide much discussion about the increased slow spindles in RBD versus controls. O'Reilly et al. [110] acknowledged the limitation of examining spindles only during NREM2, but also noteworthy is how they examined spindles in whole-night NREM2 rather than examining consecutive NREM2 sleep cycles across sleep time.

Massicotte-Marquez and colleagues [111] recruited 28 iRBD patients who met standard ICSD and PSG criteria (e.g., complaints of body movements during sleep associated with dream mentation that disrupt sleep and/or are harmful and excessive muscle activity during REM sleep) and 28 age- and sex-matched controls. The authors identified specific exclusion criteria (e.g., history of other sleep, psychiatric, and/or neurological disorders), participant instructions (e.g., no sleep-disrupting medications for at least one week, no alcohol or caffeine within 24 hrs of testing), and a standardized time in which EEG recordings began and ended. Along with other standard EEG frequencies, absolute sigma power in two bandwidths (12–14 Hz and 14–16 Hz) was examined during total NREM and the first 3 NREM cycles. No group differences in sigma power were observed. Instead, results demonstrated that iRBD patients, females in particular, had higher delta EEG (0.75–4 Hz) during NREM. The authors' discussion was focused on the delta bandwidth, and a hypothesis was made about the role of increased adenosinergic activity in the iRBD group to account for greater delta power. Of note, while the examination of slow and fast spindle activity is a study strength, the study was limited by only examining data from central (opposed to frontal and/or parietal) scalp locations and not adjusting their statistical significance in accordance with the number of analyses performed.

Finally, a study by Simor and colleagues [112] examined relative spectral power across 19 EEG derivations in a sample of young-adult chronic nightmare sufferers and age- and sex-matched controls. EEG power was examined on a 2nd recording night during NREM and REM sleep, controlling for self-reported anxiety and depression. The authors did not identify specific bandwidth categories (i.e., delta, alpha, and sigma), and instead examined spectral power in 0.25 Hz bins from 0.75–48.25 Hz. Of note, Simor et al. described a unique method to account for multiple comparisons and inflated Type I error, which they say is less conservative than commonly applied corrections and thus more optimal for relative EEG data (see [112], pg 594). Nightmare sufferers had significantly lower 1–1.25 Hz activity and higher 4–4.75 Hz and 7.75–9 Hz activity across multiple EEG derivations during whole-night NREM sleep, but no group differences were found across 0.25 Hz bins in the traditional sigma band (11–16 Hz); further, many of the results during NREM sleep were no longer significant after correcting for multiple comparisons. Relative EEG power from 10–14.5 Hz was also examined during REM sleep; however (unlike other studies reviewed above that reported examining “sigma” power during REM) Simor et al. [112] labelled this measure as “high alpha.” Relative power in this frequency band was significantly higher in the nightmare group across multiple derivations. As a follow-up (given the large number of EEG derivations), the authors compared topographic distributions of 10–14.5 Hz activity during REM sleep between groups, which revealed that the greatest difference between the two groups was evident around the left temporo-occipital area. The authors interpreted their findings during REM as reflecting a leakage of wake-like activity that interrupts sleep depth and continuity. Of note, Simor et al. [112] examined multiple EEG derivations and utilized a fast sampling rate (1024 Hz); however their study focused only on whole-night NREM and REM sleep. As such, future researchers should consider replicating this study using data from consecutive sleep cycles to elucidate potential group differences across the night.

To summarize, two of the three studies in RBD report attenuated spindle density during NREM sleep compared to controls. The first study [109] demonstrated reduced spindle density during NREM2, NREM3, and total NREM and a progressive decline in spindle density across clinical categories (i.e., lowest density in Parkinson's + RBD). The second study [110] demonstrated similar results between RBD and healthy controls, as well as the extent of variation in these differences across scalp locations, and the noteworthy divergence between slow and fast spindle activity between RBD and controls during NREM2. The third study [111] examined relative spectral power and found no group differences, despite also separating slow and fast sigma activity. However, as noted, this latter study examined data only from C3, which implies that future studies of sigma power in RBD should consider recording slow and fast spindle activity from multiple EEG derivations. Taken together, there exists some evidence for reduced spindle activity in RBD versus controls, but further study is needed to elucidate whether differences only exist in detected spindles opposed to sigma spectral power. Overall, limited empirical research examining spindle activity in RBD is available. In agreement with [110, 111], longitudinal studies of spindles in RBD versus controls are needed to examine whether progressive declines in neurological and cognitive function are associated with decreasing spindle activity across time and also to elucidate if aberrant spindle oscillations precede or follow the onset of symptoms, both of which would have implications for a better understanding and potential treatment of neurodegenerative diseases. Finally, one study of chronic nightmare sufferers was reviewed, but no group differences in sigma power were identified during NREM sleep, and differences during REM were restricted to a 10–14.5 Hz “high alpha” band. As such, more studies are needed to examine spindle activity in adults with chronic nightmares.

## 6. Bruxism

(1)* Bruxism* refers to a rhythmic clenching of the jaw occurring most often, but not exclusively, during NREM sleep [116]. Bruxism events can be tonic (sustained) or phasic (multiple bursts). Events can trigger cortical arousals from sleep, cause dental damage and pain (e.g., headaches), and be loud enough to disturb bed partners [59, 117, 118]. The pathophysiology of bruxism is not fully understood; however the disorder has been associated with neuroanatomical (e.g., brainstem structures), neurophysiological (e.g., serotonin and dopamine), and psychological pathology (e.g., stress and anxiety) [117, 118]. A study by Huynh and colleagues [119] also demonstrated that, in moderate to severe cases, bruxism events are preceded by augmented cardiac sympathetic activity.

(i) Only one study that examined spindles between participants with sleep bruxism and controls was identified. Lavigne and colleagues [58] recruited 10 bruxism participants (50% male;* M* (SD)_age_ = 27.6 (1.7) yrs) and 10 age- and sex-matched controls (*M* (SD)_age_ = 25.6 (2.4) yrs), none of whom were taking medications during the study. Following an adaptation night, the density of spindles (12–14 Hz) per hour of NREM2 sleep was determined by visual EEG scoring (128 Hz sampling rate). No group differences were found in spindle density during NREM2, or in the spindle time-course across successive NREM-REM cycles, and the authors concluded on support that bruxism patients are otherwise good sleepers. Of note, the authors examined only a narrow 12–14 Hz spindle band using visual scoring methods. Further examinations of both detected spindles and spectral EEG activity in bruxism are warranted.

## 7. Conclusions

The overarching aim of this review was to critically examine the experimental literature comparing sleep spindle oscillations between adults with different sleep disorders and healthy/good-sleeper controls. Thirty-seven studies that met stringent inclusion criteria were identified using a systematic methodology. Studies were organized across multiple diagnostic categories.

As noted at the end of Section 2 (Literature Search Strategy), four main summary points can be identified from this review. First, there is a general paucity of studies examining spindle activity among participants with NC, IH, RLS, NREM/REM parasomnias, and bruxism, compared to studies of insomnia or SDB. Additional research is needed with these other sleep-disordered groups to clarify potential group differences in spindle activity with good-sleeper controls that were observed in this review. Second, all studies employed a cross-sectional design. Chiefly, this precludes an ability to comment on whether changes in spindle oscillations might precede (i.e., precipitate) or follow/are a consequence of disturbed sleep. This highlights a need for future, longitudinal research around spindle oscillations in sleep disorders. Third, the majority of studies utilized spectral analysis techniques, which implies that less is known about spindle oscillation parameters such as density, frequency, amplitude, and duration among individuals with sleep disorders. Only 10 reviewed studies included manual and/or automatic spindle detection methods [58, 67, 82, 85–88, 107, 109, 110]. This is particularly relevant given the occasional discrepancies between visually detected and spectrally analyzed spindles. Indeed, it has been questioned whether changes in spectral power actually correspond to changes in sleep spindles [52]. Finally, discrepancies in samples (e.g., small groups and sex ratios) and study methodology within sleep disorder categories were particularly noteworthy. Throughout this review, attention has been drawn to methodological differences between studies. Such differences help explain study discrepancies and also speak to the general lack of research standardization in this area. Methodological factors most commonly inconsistent across studies include analyzed EEG derivations, EEG sampling rates, spectral analysis windows, spindle bandwidths, and the portion of sleep being examined. Overall, these differences can make it challenging to compare results for spindle dynamics between sleep-disordered groups and controls. As well, most of the studies that examined multiple EEG spectral variables (i.e., were not examining spindle activity, specifically) did not report accounting for inflated Type I error due to multiple comparisons. The tendency to conduct “shotgun,” exploratory testing of multiple EEG variables without accounting for this statistically is a noteworthy limitation in this research area.

To our knowledge, this is the first review of research examining spindle oscillations across different sleep disorders in adults. This review provides a summary of currently available research on spindle oscillations in sleep disorders and highlights gaps in knowledge implying that more research is needed to better characterize differences between sleep-disordered groups and controls. A meta-analytic examination of spindle oscillation parameters between sleep-disordered groups and controls may be warranted to better understand the potential differences in spindle activity observed in this review; however, such a meta-analysis would need to be designed carefully given the limited number of available studies and methodological differences that exist between them. Based on our observations, increased efforts to examine spindles derived from visual/automatic event-detection methods and group differences between slow and fast spindles are needed. Establishing spindle measurement and analysis guidelines will be an important step towards improving overall research standardization.

## Figures and Tables

**Table 1:** Insomnia.

Study	Sample	Demographics	Meds at Study	Measurement	Target variables	Results
Event analysis-visual						
Bastien et al., 2009 [67]	Clinical: *N* = 14 psychophysiological insomnia (PI), Control: *N* = 14	Clinical: *M*_age_ = 43.4 yr, ?% ♂ Control: *M*_age_ = 38.1 yr, 36% ♂	No	Three all-night PSGs (1 adaptation); SR = ?; spindles on C3 from nights 2 & 3	Number and density of spindles (12–14 Hz activity, >0.5 sec, 20–40 *μ*V) during NREM2	No group differences

Spectral analysis						
Freedman, 1986 [68]	Clinical: *N* = 12 idiopathic sleep-onset (SO) insomnia, Control: *N* = 12	Clinical: *M*_age_ = 31.8 (20–53) yr, 8% ♂ Control: *M*_age_ = 27.8 (19–56) yr, 33% ♂	No	Three all-night PSGs (1 adaptation); SR = 128 Hz; FFT on C3 and O1 from night 3	EEG power spectra from 1–30 Hz during the first unambiguous minute of each sleep stage	(1) No group differences during NREM; (2) insomnia group had higher 16 Hz activity during REM on O1
Merica et al., 1998 [66]	Clinical: *N* = 20 SO/sleep maintenance (SM) insomnia (*n* = 3 idiopathic, *n* = 17 PI) Control: *N* = 19	Clinical: *M*_age_ = 30.2 (20–47) yr, 40% ♂ Control: *M*_age_ = 25.3 (20–35) yr, 47% ♂	No	Three all-night PSGs (1 adaptation); SR = 128 Hz; FFT w/avg of 5 *∗* 4-sec eph on F4 from night 2	Absolute sigma power (12.5–14.75 Hz) across the first four NREM-REM cycles	(1) Insomnia group had slower sigma rise rate and attenuated power curve in NREM cycles 3 and 4; (2) insomnia group had higher sigma power curve across all 4 REM cycles
Krystal et al., 2002 [69]	Clinical: *N* = 30 persistent primary SM insomnia (*n* = 12 “SI”, *n* = 18 “OI”) Control: *N* = 20	Clinical: *M*_age_ = 54.9 (40–80) yr, 40% ♂ Control: *M*_age_ = 53.5 (40–80) yr, 40% ♂	No	One ambulatory PSG; SR = 128 Hz; FFT w/2-sec eph on C3	Absolute and relative sigma power (12.5–16 Hz) during NREM2/NREM3 and REM	(1) Higher absolute and relative sigma in SI versus OI and in OI vs. controls during NREM2/NREM3; (2) no group differences during REM
Bastien et al., 2003 [70]	Clinical 1: *N* = 15 PI, Clinical 2: *N* = 15 benzo-medicated PI, Control: *N* = 15	Clinical 1: *M*_age_ = 63.4 yr, 53% ♂ Clinical 2: *M*_age_ = 62.2 yr, 47% ♂ Control: *M*_age_ = 63.1 yr, 60% ♂	Only benzodiazepine in the medicated group	Three all-night PSGs (1 adaptation); SR = 200 Hz; FFT w/2.56-sec eph on C3 from night 2	Absolute sigma power (11.7–14.04 Hz) during the first four NREM cycles and total sleep	No group differences
Staner et al., 2003 [71]	Clinical 1: *N* = 21 PI, Clinical 2: *N* = 21 depressed PI Control: *N* = 21	Clinical 1: *M*_age_ = 40.5 yr, 52% ♂ Clinical 2: *M*_age_ = 46.5 yr, 52% ♂ Control: *M*_age_ = 44.3 yr, 52% ♂	No	Three all-night PSGs (1 adaptation); SR = 128 Hz; FFT w/2-sec eph on C3 from night 3	Spindle frequency activity (SFA: 11.5–15 Hz) for the 1st NREM cycle divided into 10 intervals, expressed as % relative to total/undivided NREM cycle	No group differences
Buysse et al., 2008 [72]	Clinical: *N* = 48 PI, Control: *N* = 25	Clinical: *M*_age_ = 30.8 yr, 40% ♂ Control: *M*_age_ = 30.6 yr, 40% ♂	No	Three all-night PSGs (1 adaptation); SR = 256 Hz; FFT w/4-sec eph on C3 and C4 from night 2	Absolute and relative sigma power (12–16 Hz) during individual NREM cycles and total NREM	(1) No overall group differences during each NREM cycle and during total NREM sleep; (2) group *∗* sex interaction, with higher absolute sigma in female insomniacs during total NREM
Forget et al., 2011 [73]	Clinical: *N* = 12 primary SM insomnia, Control: *N* = 12	Clinical: *M*_age_ = 45.4 yr, 42% ♂ Control: *M*_age_ = 44.3 yr, 42% ♂	No	Four all-night PSGs (1 adaptation); SR = 512 Hz; FFT w/1-sec eph on Fz from nights 3 & 4	Relative sigma power (12–14 Hz) before and after spontaneous and evoked K-complexes	No group differences
Spiegelhalder et al., 2012 [74]	Clinical: *N* = 25 PI (*N* = 7 “SI”, and *N* = 18 “OI”), Control: *N* = 29	Clinical: *M*_age_ = 47.8 yr, 36% ♂ Control: *M*_age_ = 46.5 yr, 38% ♂	No	One all-night PSG; SR = 200 Hz; FFT w/30-sec eph on C3	Absolute sigma power (12–16 Hz) during NREM2 and REM	(1) Total insomnia group had higher sigma during NREM2, but not during REM; (2) no group differences between SI and OI subtypes during NREM2
Israel et al., 2012 [75]	Clinical: *N* = 54 primary SM insomnia, Control: *N* = 22	Clinical: *M*_age_ = 34.6 yr, 44.4% ♂ Control: *M*_age_ = 26.5 yr, 13.6% ♂	No	Three all-night PSGs; SR = 256 Hz; FFT w/4-sec eph on C3 and C4	Mean relative sigma power (12–16 Hz) during NREM	No group differences (adjusting for age, sex)
St-Jean et al., 2013 [48]	Clinical 1: *N* = 20 SI, Clinical 2: *N* = 26 OI, Control: *N* = 21	Clinical 1: *M*_age_ = 40.6 yr, 30% ♂ Clinical 2: *M*_age_ = 41.2 yr, 46% ♂ Control: *M*_age_ = 38.6 yr, 43% ♂	No	Four all-night PSGs; SR = 512 Hz; FFT w/4-sec eph on F3, F4, Fz, C3, C4, Cz, P3, P4, Pz from nights 2 & 3	Absolute and relative sigma power (11–14 Hz) during selected portions of stable, consolidated, and artefact-free periods of NREM1–4 (“NREM”) and REM	(1) SI had higher absolute sigma versus OI on F3 and F4 during NREM; (2) SI had lower relative sigma versus OI on F3 and F4 during REM; (3) SI had lower relative sigma versus controls on all frontal and central derivations during REM; (4) SI had lower relative sigma versus OI and versus controls on F3, F4, and Fz during NREM-REM cycle 4
Wu et al., 2013 [76]	Clinical: *N* = 50 PI, Control: *N* = 32	Clinical: *M*_age_ = 36.3 yr, 50% ♂ Control: *M*_age_ = 32.7 yr, 40.6% ♂	No	Three all-night PSGs (1 adaptation); SR = 256 Hz; FFT w/4-sec eph on C3 and C4 from night 2	Absolute and relative sigma power (12–16 Hz) during the first NREM period and total NREM	No group differences
Cervena et al., 2014 [77]	Clinical 1: *N* = 10 primary SO insomnia, Clinical 2: *N* = 10 primary SM insomnia, Control: *N* = 10	Clinical 1: *M*_age_ = 34.2 yr, 70% ♂ Clinical 2: *M*_age_ = 41.6 yr, 60% ♂ Control: *M*_age_ = 41.4 yr, 50% ♂	No	Three all-night PSGs (1 night for controls); SR = 256 Hz; FFT w/15 *∗* 4-sec eph on C3 from night 2	Absolute sigma power (12–14.75 Hz) during the 5 mins before and after sleep-onset divided into 1-min intervals	No group differences
Maes et al., 2014 [78]	Clinical: *N* = 17 primary SO insomnia, Control: *N* = 11	Clinical: *M*_age_ = 36.2 (19–53) yr, 0% ♂ Control: *M*_age_ = 37.6 (21–59) yr, 0% ♂	No	Retrospective analysis of one all-night PSG; SR = 250 Hz; FFT w/4-sec eph on C4	Relative sigma power (12–16 Hz) during sleep-onset, first SWS cycle, and within 1 sec of a NREM2 K-complex	No group differences

?: unmentioned; ♂: male; Meds at Study: participants taking medication at time of sleep study; eph: epoch; sec: second; min: minute; SR: sampling rate; Hz: Hertz; PI: primary/psychophysiological insomnia; SI: subjective insomnia; OI: objective insomnia; SO: sleep-onset; SM: sleep maintenance.

**Table 2:** Hypersomnias and Disorders of Excessive Daytime Sleepiness.

Study	Sample	Demographics	Meds at Study	Measurement	Target variables	Results
*Narcolepsy-cataplexy (NC)*						
(i) Spectral analysis						
Ferri et al., 2005 [80]	Clinical: *N* = 49 NC, Control: *N* = 37	Clinical: *M*_age_ = 28.8 yr, 65% ♂ Control: *M*_age_ = 28.9 yr, 38% ♂	No	Two all-night PSGs (1 adaptation); SR = 128 or 256 Hz; FFT w/2-sec eph on C3 or C4 from night 2	Absolute and relative EEG power in 1 Hz bins from 0.5–25 Hz during NREM1–3 and REM, and for CAP measures during NREM2 and SWS	NC had higher absolute power across bins ranging within 5.5–18.5 Hz only within CAP A3 subtype during SWS, possibly reflecting arousal activity coinciding with sleep instability
Khatami et al., 2007 [81]	Clinical: *N* = 11 NC, Control: *N* = 11	Clinical: *M*_age_ = 28 (18–37) yr, 45% ♂ Control: *M*_age_ = 27 (18–41) yr, 45% ♂	No	Two all-night PSGs (1 adaptation); SR = 512 Hz; FFT w/avg of 5 *∗* 4-sec eph on C3 from night 2	Absolute sigma power (12–15 Hz) during all-night NREM and REM; time course of relative spindle frequency activity (SFA; 12–14 Hz) across and within NREM cycles	(1) No group differences in absolute power; (2) no group differences in evolution of relative SFA across NREM cycles; (3) U-shaped pattern of SFA in NC only evident within the first NREM cycle, but seen within all NREM cycles among controls

*Idiopathic hypersomnia (IH)*						
(i) Event Analysis - Visual						
Bové et al., 1994 [82]	Clinical 1: *N* = 11 idiopathic hypersomnia (IH), Clinical 2: *N* = 10 narcolepsy, Control: *N* = 11	Clinical 1: *M*_age_ = 46 (16–67) yr, ?% ♂ Clinical 2: *M*_age_ = 45 (17–76) yr, ?% ♂ Control: *M*_age_ = 41.5 (11–70) yr, ?% ♂	No	One all-night PSG; SR = ? Hz; C3, C4, and O1-O2	Bilateral spindle density (12–14 Hz, >0.5 sec, >20 *μ*V) calculated in 10-min segments of NREM2 and averaged; time-course of spindle density across each hour of sleep	(1) Total clinical group had greater overall mean spindle density versus controls, especially at the beginning and end of sleep time; (2) spindle density greater in IH versus narcolepsy only in the middle hours of sleep time; (3) spindle density in IH did not decline across the night as in other groups
(ii) Spectral Analysis						
Sforza et al., 2000 [83]	Clinical: *N* = 10 IH, Control: *N* = 10	Clinical: *M*_age_ = 25.4 (18–40) yr, 40% ♂ Control: *M*_age_ = 25.2 (19–40) yr, 40% ♂	No	One all-night PSG; SR = 128 Hz; FFT w/avg of 5 *∗* 4 sec-eph on C3	Absolute sigma power (12.25–15 Hz) during the first four NREM-REM cycles	No group differences
Pizza et al., 2013 [84]	Clinical 1: *N* = 19 IH, Clinical 2: *N* = 17 NC; Control: *N* = 13	Clinical 1: *M*_age_ = 46 yr, 68% ♂ Clinical 2: *M*_age_ = 45 yr, 77% ♂ Control: *M*_age_ = 44.9 yr, 54% ♂	No	Two all-night PSGs (1 adaptation); SR = 128 Hz; FFT w/2-sec eph on C3 or C4 from night 2	Absolute and relative sigma power (11.5–15.5 Hz) during NREM2, SWS, and REM divided into 4 *∗* 90-min periods	(1) Overall group effect (NC > IH > controls) for absolute sigma during REM in the 3rd 90-min period; (2) overall group effect (NC > IH > controls) for relative sigma during whole-night SWS, and during SWS and REM in the 2nd 90 min period

*Sleep disordered breathing*						
(i) Event analysis-visual						
Himanen et al., 2003 [85]	Clinical: *N* = 12 obstructive sleep apnea (OSA), Control: *N* = 12	Clinical: Mdn_age_ = 48 yr, 50% ♂ Control: Mdn_age_ = 43 yr, 50% ♂	No	Two all-night PSGs (1 adaptation); SR = 100 Hz (minimum); spindles (using FFT w/1-sec eph) from left (Fp1, C3, O1) & right leads (Fp2, C4, O2) from night 2	Mdn number and density of bilateral spindles on frontal, central, and occipital derivations; Mdn spindle frequency across 5 NREM cycles, each divided into 10 equal segments	(1) No group differences in number and density of spindles; (2) OSA had almost consistently slower spindle frequencies, with 30–70% of 10 equal segments among each NREM cycle showing significant differences
(ii) Event analysis-automatic						
Huupponen et al., 2003 [86]	Clinical: *N* = 10 OSA, Control: *N* = 7	Clinical: Mdn_age_ = 48.5 (34–66) yr, 40% ♂ Control: Mdn_age_ = 45 (31–63) yr, 40% ♂	?	Two all-night PSGs; SR = 200 Hz; auto spindle detection from power spectra on C3 and C4 from night 2	Number of detected spindles (9.8–16 Hz), and the evolution of spindle frequencies across and within individual sleep depth cycles	(1) No group differences in the number of detected spindles; (2) mean spindle frequency lower in OSA (11.7 Hz) than controls (12.5 Hz) across the night and within sleep depth cycles 3–5
Ondze et al., 2003^a^ [87]	Clinical: *N* = 18 mild SDB Control: *N* = 18	Clinical: *M*_age_ = 35.5 (18–56) yr, 67% ♂ Control: *M*_age_ = 33.1 (18–52) yr, 39% ♂	No	Three all-night PSGs (1 adaptation); SR = 128 Hz; integrated digital filter analysis on C3 and C4 from night 2	Sleep spindle index (SSI; 13–17 Hz) during total sleep, and in each of NREM2, NREM3, and REM sleep, both overall and across consecutive sleep cycles	SSI lower in SDB versus controls (a) during total sleep, (b) within each of four consecutive NREM-REM sleep cycles, and (c) during each of NREM2 and NREM3 examined both overall and across consecutive sleep cycles
Carvalho et al., 2014 [88]	Clinical 1: *N* = 11 mild OSA, Clinical 2: *N* = 10 moderate OSA, Control: *N* = 7	Clinical 1: *M*_age_ = ? yr, ?% ♂ Clinical 2: *M*_age_ = ? yr, ?% ♂ Control: *M*_age_ *=* ? yr, ?% ♂	Yes	One all-night PSG; SR = 256 Hz; Auto spindle analysis on frontal (F3 + F4), central (C3 + C4), and parietal (P3 + P4) leads	Frequency variations (“chirp”) of spindles (11–16 Hz, divided into (<13 Hz) and fast (≥13 Hz) types) measured from 3 *∗* 10-min segments of NREM2	(1) Moderate OSA had a decreased percentage of decelerating spindles among frontal and parietal slow spindles versus mild OSA and versus controls, but not between mild OSA and controls; (2) moderate OSA had a loss of spindle deceleration (i.e., decline in Hz/time) versus mild OSA and versus controls among parietal slow spindles
(iii) Spectral analysis						
Guilleminault et al., 2001 [89]	Clinical: *N* = 36 (*N* = 12 each in groups of upper airway resistance; OSA; and hypopnea), Control: *N* = 12	Clinical: *M*_age_ = 42–43.5 (30–54.3) yr; 100% ♂ Control: *M*_age_ = 41.9 (30.3–54.2) yr. 100% ♂	?	One all-night PSG; SR = 128 Hz; FFT w/avg of 6 *∗* 4-sec eph on C3	Absolute sigma power (12.25–15 Hz; overall and in 1 Hz bins) in 5-min segments averaged across total NREM-REM sleep, and both NREM and REM separately	(1) Total clinical group had higher sigma (12.25–15 Hz) power versus controls during total sleep, but no differences among clinical subgroups reported; (2) all clinical groups had lower 13-14 Hz activity versus controls during NREM, but no differences between clinical subgroups reported; (3) no group differences during REM
Dingli et al., 2002 [90]	Clinical: *N* = 15 OSA Control: *N* = 7	Clinical: *M*_age_ = 51 yr, 93% ♂ Control: *M*_age_ = 50 yr, 57% ♂	No	One all-night PSG; SR = 100 Hz; FFT w/6 *∗* 4-sec eph on C4	Relative sigma power (12–16 Hz) during NREM, REM, and Total sleep (both with and without surrounding respiratory events)	(1) No between-group differences; (2) higher sigma power during NREM and total sleep within OSA group after, versus before, individual respiratory events, particularly those with associated arousals
Ondze et al., 2003^a^ [87]	Clinical: *N* = 18 mild SDB Control: *N* = 18	Clinical: *M*_age_ = 35.5 (18–56) yr, 67% ♂ Control: *M*_age_ = 33.1 (18–52) yr, 39% ♂	No	Three all-night PSGs (1 adaptation); SR = 128 Hz; FFT w/avg of 5 *∗* 4-sec eph on C3 and C4 from night 2	Absolute sigma power (0.25 Hz bins from 12.25–15 Hz) across consecutive NREM-REM sleep cycles and during total sleep	SDB had lower sigma power versus controls across each of four consecutive NREM-REM sleep cycles and during total (whole-night) NREM-REM sleep
Chervin et al., 2005 [91]	Clinical: *N* = 27 SDB Control: *N* = 11	Clinical: *M*_age_ = 45.2 yr, 67% ♂ Control: *M*_age_ = 33.2 yr, 18% ♂	No	One all-night PSG; SR = 128 Hz; EEG power derived using 5th-order Butterworth filter on C3	Relative sigma power (13–15 Hz) during respiratory cycles from the first 3 hours of sleep, with each respiratory cycle divided into 4 segments	No group differences
Xavier et al., 2007 [92]	Clinical: *N* = 13 OSA Control: *N* = 14	Clinical: *M*_age_ = 49.1 yr, 54% ♂ Control: *M*_age_ = 46.2 yr, 57% ♂	?	One all-night PSG; SR = 100 Hz; Welch method on C3	Relative sigma power (12–16 Hz) during total sleep and in the 10 sec before and after respiratory events	No group differences
Abdullah et al., 2010 [93]	Clinical: *N* = 11 OSA Control: *N* = 8	Clinical: *M*_age_ = 50.6 yr, 82% ♂ Control: *M*_age_ = 48.1 yr, 63% ♂	?	One all-night PSG; SR = 256 Hz; FFT w/average of 10 *∗* 30-sec eph on C3	Relative sigma power (13–16.5 Hz) before sleep, and across NREM1–4 and REM	OSA had lower sigma during NREM2

?: unmentioned; ♂: male; Meds at Study: participants taking medication at time of sleep study; eph: epoch; sec: second; min: minute; SR: sampling rate; Hz: Hertz; Mdn: median; NC: narcolepsy-cataplexy; IH: idiopathic hypersomnia; OSA(/H): obstructive sleep apnea(/hypopnea).

^a^Study is listed twice for providing both event analysis and spectral analysis data.

**Table 3:** Sleep-Related Movement Disorders/Parasomnia.

Study	Sample	Demographics	Meds at Study	Measurement	Target variables	Results
*Restless Legs Syndrome*						
(i) Spectral analysis						
Hornyak et al., 2005 [105]	Clinical: *N* = 20 idiopathic restless legs syndrome (RLS), Control: *N* = 20	Clinical: *M*_age_ = 50.4 yr, 50% ♂ Control: *M*_age_ = 51.8 yr, 50% ♂	No	One all-night PSG; SR = 200 Hz; FFT w/30-sec eph on C3	Absolute sigma power (12–16 Hz) during total NREM and REM sleep, and in NREM2 and REM epochs with (a) arousals, (b) leg movements, and (c) leg movements + arousals	No group differences
Ferri et al., 2014 [106]	Clinical 1: *N* = 27 idiopathic RLS, Clinical 2: *N* = 11 chronic primary insomnia, Control: *N* = 14	Clinical 1: *M*_age_ = 53.6 yr, 56% ♂ Clinical 2: *M*_age_ = 58.9 yr, 45% ♂ Control: *M*_age_ = 50.3 yr, 50% ♂	No	Two all-night PSGs (1 adaptation); SR = 128 Hz; FFT w/4-sec eph on C3 or C4 from night 2	Absolute and relative sigma power (11.75–14.75 Hz) in the 5 min before and after sleep-onset period (SOP)	No group differences

*NREM parasomnia*						
(i) Event Analysis-automatic						
Espa et al., 2000^a^ [107]	Clinical: *N* = 11 w/sleepwalking (SW) and/or sleep terrors, Control: *N* = 11	Clinical: *M*_age_ = 31.2 (22–40) yr, 45% ♂ Control: *M*_age_ = 32.1 yr, 45% ♂	No	Two all-night PSGs (1 adaptation); SR = 128 Hz; Integrated digital filtering analysis on C3 and C4 from night 2	Sleep spindle index (SSI; 13–17 Hz) across consecutive NREM-REM sleep cycles, and during each of NREM2, NREM3, and REM sleep both overall and in consecutive sleep cycles	(1) SSI lower in PA versus controls during the 2nd and 4th NREM-REM sleep cycle; (2) SSI lower in PA vs. controls during total NREM3, and during the 2nd and 4th NREM3 cycle
(ii) Spectral analysis						
Espa et al., 2000^a^ [107]	Clinical: *N* = 11 w/sleepwalking (SW) and/or sleep terrors, Control: *N* = 11	Clinical: *M*_age_ = 31.2 (22–40) yr, 45% ♂ Control: *M*_age_ = 32.1 yr, 45% ♂	No	Two all-night PSGs (1 adaptation); SR = 128 Hz; FFT w/avg of 5 *∗* 4-sec eph on C3 and C4 from night 2	Absolute sigma power (13–17 Hz) during total NREM and REM sleep; relative power in the 2nd, 3rd, and 4th NREM and REM cycles, expressed as a percentage of power in the 1st cycle	No group differences
Guilleminault et al., 2006 [108]	Clinical: *N* = 10 SW, Control: *N* = 10	Clinical: *M*_age_ = 25.6 yr, 70% ♂ Control: *M*_age_ = 25.8 yr, 70% ♂	?	One all-night PSG; SR = 256 Hz; FFT w/avg of 15 *∗* 4-sec eph on C4	Relative sigma power (12.25–16 Hz) during total NREM	No group differences

*REM parasomnias*						
(i) Event Analysis-automatic						
Christensen et al., 2014 [109]	Clinical 1: *N* = 15 idiopathic REM behaviour disorder (iRBD), Clinical 2: *N* = 15 Parkinson's + RBD (PD + RBD), Clinical 3: *N* = 15 Parkinson's without RBD (PD − RBD) Control: *N* = 15	Clinical 1: *M*_age_ = 60.1 yr, 80% ♂ Clinical 2: *M*_age_ = 62.4 yr, 73% ♂ Clinical 3: *M*_age_ = 61.9 yr, 53% ♂ Control: *M*_age_ = 58.3 yr, 40% ♂	Only dopamine-acting meds allowed	One all-night PSG; SR = ? Hz; Auto spindle scoring on F3, C3	Sleep spindle (11–16 Hz) density during total NREM, NREM1–4 separately, and REM	(1) PD + RBD and iRBD had lower spindle density versus controls in NREM2, NREM3, and total NREM (*p* < 0.01), while PD-RBD had lower spindle density versus controls only during NREM2 and total NREM (*p* < 0.05) (PD + RBD < iRBD < PD − RBD < controls); (2) no group differences during REM
O'Reilly et al., 2015 [110]	Clinical: *N* = 35 RBD, Control: *N* = 35	Clinical: *M*_age_ = 63.4 yr, 80% ♂ Control: *M*_age_ = 63.4 yr, 80% ♂	?	? all-night PSG; SR = 256 Hz; auto spindle scoring on 19 EEG sites using data pooled from various studies/assessments	Sleep spindle (11–16 Hz) mean density and topographical distribution during NREM2	(1) RBD had 6–35% lower overall spindle density maximal on central and parietal derivations; (2) RBD had lower fast spindle density in some frontal, and all examined central, parietal, temporal, and occipital derivations; (3) RBD had higher slow spindle density in F3/4, C3/4, P3/4, O1/2, and T3/5/6
(ii) Spectral analysis						
Massicotte-Marquez et al., 2005 [111]	Clinical: *N* = 28 idiopathic RBD, Control: *N* = 28,	Clinical: *M*_age_ = 66.6 (52–77) yr, 75% ♂ Control: *M*_age_ = 66.3 (51–81) yr, 75% ♂	No	One all-night PSG; SR = 256 Hz; FFT w/avg of 5 *∗* 4-sec eph on C3	Absolute sigma1 (12–14 Hz) and sigma 2 (14–16 Hz) power for total NREM and for the first three NREM cycles	No group differences
Simor et al., 2013 [112]	Clinical: *N* = 19 with nightmares (NM) Control: *N* = 21	Clinical: *M*_age_ = 20.87 yr, 53% ♂ Control: *M*_age_ = 21.57 yr, 52% ♂	No	Two all-night PSGs (1 adaptation); SR = 1024 Hz; FFT w/4-sec eph across 19 EEG derivations from night 2	Relative spectral power in 0.25 Hz bins during whole-night NREM, and “high alpha” (10–14.5 Hz) during REM	(1) No group differences in sigma power during NREM; (2) NM had higher 10–14.5 Hz “high alpha” activity during REM across scalp regions, especially over the left temporo-occipital area

?: unmentioned; ♂: male; Meds at Study: participants taking medication at time of sleep study; eph: epoch; sec: second; min: minute; SR: sampling rate; Hz: Hertz; RLS: restless legs syndrome; SW: sleepwalkers; NM: nightmare sufferers; PA: combined parasomnia group; RBD: REM behaviour disorder; PD + RBD: Parkinson's disease with RBD.

^a^Study is listed twice for providing both event analysis and spectral analysis data.

## Additional Points

References [48, 58, 66–78, 80–93, 105–112] indicate experimental studies included in the systematic review.
